# A multiscale approximation in a heat shock response model of E. coli

**DOI:** 10.1186/1752-0509-6-143

**Published:** 2012-11-21

**Authors:** Hye-Won Kang

**Affiliations:** 1 Mathematical Biosciences Institute, Ohio State University, Columbus, OH, USA

**Keywords:** Multiscale, Markov chains, Chemical reaction, Reaction networks, Heat shock

## Abstract

**Background:**

A heat shock response model of *Escherichia coli* developed by Srivastava, Peterson, and Bentley (2001) has multiscale nature due to its species numbers and reaction rate constants varying over wide ranges. Applying the method of separation of time-scales and model reduction for stochastic reaction networks extended by Kang and Kurtz (2012), we approximate the chemical network in the heat shock response model.

**Results:**

Scaling the species numbers and the rate constants by powers of the scaling parameter, we embed the model into a one-parameter family of models, each of which is a continuous-time Markov chain. Choosing an appropriate set of scaling exponents for the species numbers and for the rate constants satisfying balance conditions, the behavior of the full network in the time scales of interest is approximated by limiting models in three time scales. Due to the subset of species whose numbers are either approximated as constants or are averaged in terms of other species numbers, the limiting models are located on lower dimensional spaces than the full model and have a simpler structure than the full model does.

**Conclusions:**

The goal of this paper is to illustrate how to apply the multiscale approximation method to the biological model with significant complexity. We applied the method to the heat shock response model involving 9 species and 18 reactions and derived simplified models in three time scales which capture the dynamics of the full model. Convergence of the scaled species numbers to their limit is obtained and errors between the scaled species numbers and their limit are estimated using the central limit theorem.

## Background

Stochasticity may play an important role in biochemical systems. For example, stochasticity may be beneficial to give variability in gene expression, to produce population heterogeneity, and to adjust or respond to fluctuations in environment [1]. We are interested in local dynamics of biochemical networks involving some species with a small number of molecules so that the system is assumed to be well-mixed and relative fluctuations of small species numbers may play a role in the system dynamics.

The conventional stochastic model for the well-stirred biochemical network is based on the chemical master equation. The chemical master equation governs the evolution of the probability density of species numbers and is expressed as the balanced equation between influx and outflux of the probability density. When the biochemical network involves many species or bimolecular reactions, it is rarely possible to obtain an exact solution of the master equation in a closed form. Instead of searching for the solution of the master equation, stochastic simulation algorithms are used to obtain the temporal evolution of the species numbers. For example, Gillespie’s Stochastic Simulation Algorithm (SSA, or the direct method) is well known [2,3] and provides a realization of the exact trajectory of the sample path for the species numbers. As the biochemical network has more species and reactions, SSA becomes computationally expensive and more efficient algorithms were suggested by many authors [4-6]. The detailed review of stochastic simulation methods, stochastic approximations, and hybrid simulation methods is given in [7]. For models with well-separated time scales, numerous authors suggested stochastic simulation algorithms for biochemical reaction networks by assuming that “fast” subnetworks have reached a “partial equilibrium” [6] or a “quasi-steady state” [4]. Using these assumptions, the approximate stochastic simulation algorithms involve a reduced number of species or reactions.

On the other hand, Ball et al. [8] described the state of the biochemical reaction network in the well-stirred system directly using stochastic equations for species numbers, and suggested an approximation of the reaction network via limiting models derived using different scalings for the species numbers and for the reaction rate constants. Kang and Kurtz [9] extended this multiscale approximation method and gave a systematic way to obtain limiting models in the time scales of interest. Conditions are given to help identify appropriate values for a set of scaling exponents which determine the time scale of each species and reaction. Using this method, nonstationary behavior of biochemical systems can be analyzed. Moreover, application of the method is flexible in the sense that the method does not require the exact parameter values but gives approximations valid for a range of parameter values. More recently, Crude et al. [10] also proposed a reduction method to derive simplified models with preserving stochastic properties and with key parameters using averaging and hybrid simplification.

The multiscale approximation method in [9] requires consideration of magnitude of both species numbers and rate constants of the reactions involving the corresponding species. When a moderately fast reaction involves two species, one with a small number of molecules and the other with a large number of molecules, the effects of this reaction on these species are different. Net molecule changes of species with large numbers due to the reaction is less noticeable than those of species with small numbers. Therefore, though the same reaction governs these species, their time scales may be different from each other. Letting *N*_0_ be a fixed constant and choosing a large value for *N*_0_, for example *N*_0_=100, we express magnitudes of species numbers and reaction rate constants in terms of powers of *N*_0_ with different scaling exponents. For instance, 1 to 10molecules are expressed as $1\times N_{0}^{0}$ to $10\times N_{0}^{0}\text{molecules}$, 500 to 800molecules are rewritten as 5×*N*_0_ to 8×*N*_0_molecules, and 0.0002 sec becomes $2\times N_{0}^{-2}\sec$. Assuming *N*_0_ is large, we replace *N*_0_by a large parameter *N* and stochastic equations for species numbers are expressed in terms of *N*. Then, *N* is an analogue of 1/*ε* where *ε* is a small parameter in perturbation theory.

A specific time scale of interest is expressed in terms of a power of *N*, and its exponent contributes to reaction rates due to change of variables in time. For each species (or linear combination of species), we compare a power of *N* for the species number and those for reaction rates involving this species. Consider a case when the power for the species number is larger than those for the rates of all reactions where the species is involved. Then net molecule changes due to the reactions are not large enough to be noticeable in this time scale, and the species number is approximated as constant. Next, consider a case when the power for the species number is smaller than those for some reaction rates involving the species. In this case, the species number fluctuates very rapidly due to the fast reactions in this time scale, and the averaged behavior of the species number can be described in terms of other species numbers. The method of averaging is similar to approximation of one variable in terms of others using a quasi-steady state assumption. Last, when the power for the species number is equal to those for the rates of reactions where the species is involved, the scaled species number is approximated by a nondegenerate limit describing nonstationary behavior of the species number in the specific time scale of interest. The limit could be described in various kinds of variables: a continuous time Markov chain, a deterministic model given by a system of ordinary differential equations, or a hybrid model with both discrete and continuous variables. Since some of the scaled species numbers are approximated as constants or the averaged behavior of some species numbers is expressed in terms of other variables, dimension of species in the approximation of the biochemical network is reduced.

In the multiscale approximation method, scaling exponents for species numbers and for reaction rate constants are not uniquely determined, since the choice of values for the exponents is flexible. For example, 0.005 sec can be expressed as $0.5\times N_{0}^{-1}$ or $5\times N_{0}^{-1.5}$ when *N*_0_=100. The goal in this method is to find an appropriate set of scaling exponents to obtain a nondegenerate limit of the scaled species numbers. Orders of magnitude of species numbers in the propensities affect reaction rates, and reaction rates contribute to determining rates of net molecule changes of the species involved in the reactions. Since species numbers and reaction rates interact, it is not easy to determine scaling exponents for all species numbers and reaction rate constants so that the limits of the scaled species numbers become balanced.

Kang and Kurtz [9] introduced *balance conditions* for the scaling exponents, which help to determine values for a set of exponents. The key idea in these conditions is that for each species (or linear combination of species) the maximum of scaling exponents in the rates of the reactions where this species is produced should be the same as that in the rates of the reactions where this species is consumed, i.e. maximal production and consumption rates of the species should be balanced in the order of magnitude. In case the maximums of scaling exponents for productions and consumptions are not balanced for some species, an increase or decrease of the scaled species number can be described by its limit during a certain time period. However after this time period, the scaled species number will either become zero or blow up to infinity. Therefore, if some of the scaled species numbers are not balanced due to a difference between orders of magnitude of production and consumption rates, the chosen scaling is valid up to a certain time scale. After this time scale, we need to choose different values for scaling exponents. In each time scale of interest we derive a limiting model including a subset of species and reactions, which is used to approximate the state of the full reaction network. The multiscale approximation method is applicable in case some of reaction rates are not known accurately, since the chosen scaling is applicable in some ranges of the parameters. Therefore, based on the behavior of the limiting models, we may be able to estimate behavior for a range of parameter values without performing a huge number of stochastic simulations.

**Figure 1:**
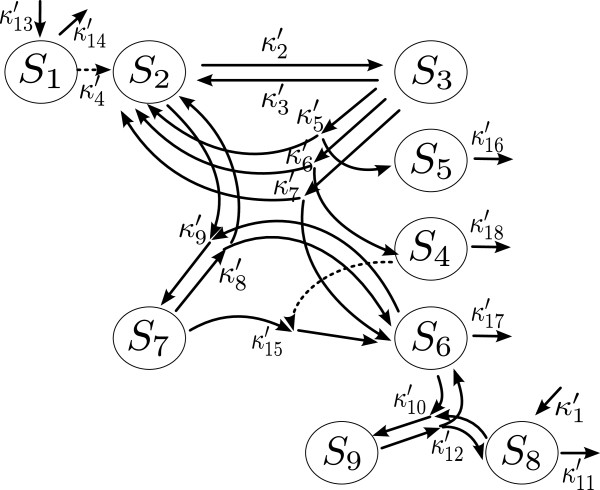
**A chemical reaction network in the heat shock response model of E. coli.** A dotted line represents the effect of the species acting as catalysts. $\kappa_{k}^{\prime }$’s represent stochastic reaction rate constants.

The paper [9] included several simple examples of biochemical networks involving two to four species, and derived limiting models in each time scale of interest. To apply this method, more scaling exponents must be determined as the biochemical network involves more species or reactions. Therefore, it is challenging to apply the method to complex biochemical systems and to determine appropriate values for scaling exponents so that the corresponding limiting models preserve important dynamical features of the full system. One of the goals of this paper is to illustrate how to apply this method to an example with significant complexity. In this paper, using a significantly complicated biochemical network, we derive limiting models, show convergence of the scaled species numbers to their limit, and estimate the error analytically between the scaled species numbers and their limit. We analyze a heat shock response model of *Escherichia coli* (*E. coli*) developed by Srivastava, Peterson, and Bentley in [11]. The model involves 9 species and 18 reactions with significant complexity as shown in Figure 1, and it has various time scales due to wide ranges of species numbers and reaction rate constants. Because of various scales involved, this model has been used as an example to show accuracy of the stochastic simulation algorithms which are developed to increase computational efficiency using the multiscale nature of the chemical reaction network [12,13]. Another version of a heat shock response model of *E. coli* is studied in [6] using an accelerated SSA that also exploits the multiscale nature of the system.

Applying the multiscale approximation method to the heat shock response model of *E. coli*, we derive limiting models in three time scales of our interests, which approximate the full network given in Figure 1. Denote *∅* as species we are not interested in. Let *S*_*i*_ represent the *i*th species and *S*_23_be addition of species *S*_2_and *S*_3_. *A*→*B* denotes a reaction where one molecule of species *A* is converted to one molecule of species *B*. In the early stage of time period of order 1 sec, we obtain the following reduced network:

$$\begin{array}{lll} & \emptyset \to S_{2}⇌S_{3},\; & \;\\ & \emptyset \to S_{8}.\; & \; \end{array}$$

The reduced network in the early stage has very simple structure without any bimolecular reactions, and all reactions involved are either production from a source or conversion. Moreover, the reduced network is well separated into two due to independence of *S*_8_from *S*_2_and *S*_3_.

In the medium stage of time period of order 100 sec, the full network is reduced to

$$\begin{array}{lll} & \emptyset \to S_{23},\; & \;\\ & \emptyset \to S_{6}\overset{S_{8}}{\to }\emptyset ,\;\emptyset \overset{S_{23}}{\to }S_{6},\; & \;\\ & S_{7}\to S_{6},\; & \;\\ & \emptyset \to S_{8},\; & \; \end{array}$$

where a species over the arrow accelerates or inhibits the corresponding reaction. The reaction does not change this species number, but the propensity of the corresponding reaction is a function of this species number. In this time scale, conversion between *S*_2_ and *S*_3_ occurs very frequently and *S*_2_and *S*_3_play a role as a single “virtual” species rather than separate species. The species numbers of *S*_23_ and *S*_8_are described as two independent birth processes and the species number of *S*_7_ is governed by conversion. In this time scale, the species number of *S*_8_is normalized and treated as a continuous variable. The interesting thing is that the behavior of the species *S*_8_ which rapidly increases in time is well approximated in both first and second time scales.

In the late stage of time period of order 10,000 sec, we get a reduced network with more species involved than those in the previous time scales. However, the reduced network is still much simpler than the full network in Figure 1. At this time scale, we get

$$\begin{array}{lll} & \emptyset \to S_{1}\to \emptyset ,\; & \;\\ & \emptyset \overset{S_{1}}{\to }S_{23}\overset{S_{8},S_{9}}{\to }\emptyset ,\; & \;\\ & \emptyset \overset{S_{23}}{\to }S_{4}\to \emptyset ,\; & \;\\ & \emptyset \overset{S_{23}}{\to }S_{5}\to \emptyset ,\; & \;\\ & \emptyset \to S_{8}\to \emptyset ,\;S_{8}\overset{S_{23}}{\to }\emptyset ,\; & \;\\ & \emptyset \overset{S_{23}}{\to }S_{9}.\; & \; \end{array}$$

As we see in Figure 1, the full network involves reactions with more than two reactants or products. However, all reactions in the reduced network at the times of order 10,000 sec consist of either production or degradation of each species, though most of the species (6 species out of 9) are involved in the reduced model. As in the medium stage of time period, *S*_2_and *S*_3_play a role as a single species. In the early and medium stages of time period propensities are in a form following the law of mass action, while in the late stage of time period the propensity for degradation of *S*_23_ is a nonlinear function of the species numbers similar to the reaction rate appearing in the Michaelis-Menten approximation for an enzyme reaction. The nonlinear function involves the species numbers of *S*_23_, *S*_8_, and *S*_9_, which come from averaging of the species numbers of *S*_2_and *S*_6_which fluctuate rapidly in the third time scale. Similarly, the propensity of catalytic degradation of *S*_8_ is not proportional to the number of molecules of *S*_8_.

In the late stage of time period of order 10,000 sec, we study the error between the scaled species numbers and their limit analytically using the central limit theorem derived in [14] and show that the error is of order 10^−1^.

## Methods

In the next several sections, we apply the multiscale approximation to the heat shock response model of *E. coli* and derive the limiting models. The multiscale approximation method is described in terms of the following steps so that the method can be applied to the general cases. 

1. Write a chemical reaction network involving *s*_0_species and *r*_0_ reactions in the form of

$$\overset{s_{0}}{\underset{i=1}{\sum }}\nu_{\mathrm{ik}}S_{i}\to \overset{s_{0}}{\underset{i=1}{\sum }}\nu_{\mathrm{ik}}^{\prime }S_{i},\;k=1,\cdots \;,r_{0},$$

where *ν*_*ik*_ and $\nu_{\mathrm{ik}}^{\prime }$ are nonnegative integers. Rearrange the reactions so that the reaction rate constants are decreasing monotonically as *k* gets large.

2. Derive a system of stochastic equations for species numbers. 

(a) Letting *X*_*i*_(*t*) be the number of molecules of species *S*_*i*_at time *t*, the corresponding stochastic equation is

$$X_{i}(t)=X_{i}(0)+\overset{r_{0}}{\underset{k=1}{\sum }}R_{k}^{t}(\lambda_{k}(X))(\nu_{\mathrm{ik}}^{\prime }-\nu_{\mathrm{ik}}),\;i=1,\cdots s_{0},$$

where $R_{k}^{t}(\cdot )$ counts the number of times that the *k*th reaction occurs up to time *t*.

(b) *λ*_*k*_(*x*) is determined by a stochastic version of mass action kinetics, and is expressed as a product of the rate constant and the numbers of molecules of reactants. If the *k*th reaction is second-order ($\overset{s_{0}}{\underset{i=1}{\sum }}\nu_{\mathrm{ik}}=2$) with different types of reactants, $\lambda_{k}(x)=\kappa_{k}^{\prime }x_{p}x_{q}$. When the reactants are two molecules of the same species, $\lambda_{k}(x)=\kappa_{k}^{\prime }x_{p}(x_{p}-1)$.

3. Derive a system of stochastic equations for the normalized species numbers after a time change, *Z*^*N*,*γ*^(*t*). 

(a) In the equation for *X*_*i*_(*t*) obtained in Step 2 (a), replace *X*_*i*_by $Z_{i}^{N,\gamma }$ and divide reaction terms by *N*^*α*^_*i*_. In the *k*th reaction term, put *N*^*γ* + *ρ*^_*k*_ in the propensity and replace *λ*_*k*_(*X*) by ${\hat{\lambda }}_{k}(Z^{N,\gamma })$. Then, we have

$$\begin{array}{l} Z_{i}^{N,\gamma }(t)=Z_{i}^{N,\gamma }(0)+N^{-\alpha_{i}}\overset{r_{0}}{\underset{k=1}{\sum }}R_{k}^{t}(N^{\gamma +\rho_{k}}{\hat{\lambda }}_{k}(Z^{N,\gamma }))\\ \;\times (\nu_{\mathrm{ik}}^{\prime }-\nu_{\mathrm{ik}}),\;i=1,\cdots s_{0}. \end{array}$$

(b) In the equation in Step 3 (a), $\rho_{k}=\beta_{k}+\overset{s_{0}}{\underset{j=1}{\sum }}\alpha_{j}\nu_{\mathrm{jk}}$.

(c) In the most reactions, ${\hat{\lambda }}_{k}$ is obtained by replacing $\kappa_{k}^{\prime }$ by *κ*_*k*_in *λ*_*k*_. In case the *k*th reaction is second-order with reactants of the same species, $\lambda_{k}(x)=\kappa_{k}^{\prime }x_{p}(x_{p}-1)$ is replaced by ${\hat{\lambda }}_{k}(z)=\kappa_{k}z_{p}(z_{p}-N^{-\alpha_{p}})$.

4. Write a set of species balance equations and their time-scale constraints. 

(a) Define $\Gamma_{i}^{+}$ and $\Gamma_{i}^{-}$ as subsets of reactions where the species number of *S*_*i*_increases or decreases every time the reaction occurs. Comparing *ρ*_*k*_’s for $k\in \Gamma_{i}^{+}$ and those for $k\in \Gamma_{i}^{-}$, set the balance equations as

$$\underset{k\in \Gamma_{i}^{+}}{\max}\rho_{k}=\underset{k\in \Gamma_{i}^{-}}{\max}\rho_{k},\;i=1,\cdots \;,s_{0}.$$

(b) Time-scale constraints are given as

$$\gamma \leq \underset{k\in \Gamma_{i}^{+}\cup \Gamma_{i}^{-}}{\max}\rho_{k},\;i=1,\cdots \;,s_{0}.$$

5. Find a minimum set of linear combinations of species whose maximum of collective production (or consumption) rates may be different from that of one of any species. We construct a minimum set of linear combinations of species by selecting a linear combination of species if any reaction term involving the species consisting of the linear combination is canceled in the equation for the linear combination of species.

6. For each selected linear combination of species, write a collective species balance equation and its time-scale constraint. They are obtained similarly to the ones in Step 4 using subsets of reactions where the number of molecules of linear combinations of species either increases or decreases instead of using $\Gamma_{i}^{+}$ and $\Gamma_{i}^{-}$.

7. Select a large value for *N*_0_and choose an appropriate set of *α*_*i*_’s and *β*_*k*_’s so that 

(a) the species number *X*_*i*_and the reaction rate constant $\kappa_{k}^{\prime }$ are approximately of orders $N_{0}^{\alpha_{i}}$ and $N_{0}^{\beta_{k}}$;

(b) the normalized species number $Z_{i}^{N,\gamma }$ and the scaled reaction rate constant *κ*_*k*_are of order 1;

(c) most of the balance equations obtained in Steps 4 and 6 are satisfied;

(d) *β*_*k*_’s are monotone decreasing among each class of reactions which have the same number of molecules of reactants.

8. Plugging the chosen values for *α*_*i*_’s and *β*_*k*_’s in the time-scale constraints obtained in Steps 4 and 6, compute an upper bound (denoted as *γ*_0_) for a time-scale exponent. Then, the chosen set of exponents *α*_*i*_’s and *β*_*k*_’s can be used for *γ*satisfying *γ*≤*γ*_0_. For *γ*>*γ*_0_, select another set of exponents *α*_*i*_’s and *β*_*k*_’s using Steps 7 and 8.

9. Using each set of values for *α*_*i*_’s and *β*_*k*_’s, identify a natural time scale exponent of each species (denoted as *γ*_*i*_ for species *S*_*i*_) so that *γ*_*i*_ satisfies

$$\underset{k\in \Gamma_{i}^{+}\cup \Gamma_{i}^{-}}{\max}(\gamma_{i}+\rho_{k})\;\;=\;\;\alpha_{i},\;i=1,\cdots \;,s_{0}.$$

We collect *γ*_*i*_’s with the same values, whose species are in the same time scales in the approximation.

10. Modify *α*_*i*_’s and *β*_*k*_’s so that the conditions in Step 7 are satisfied and that *γ*_*i*_’s are divided into appropriate number of values, which gives the number of time scales, *N*^*γ*^=*N*^*γ*^_*i*_, we are interested in.

11. For each chosen *γ*, derive a limiting equation for each species *S*_*i*_with *γ*_*i*_=*γ*. Using the stochastic equation obtained in Step 3 (a), we let *N* go to infinity. 

(a) For $k\in \Gamma_{i}^{+}\cup \Gamma_{i}^{-}$, the *k*th reaction term converges to zero if *α*_*i*_>*γ* + *ρ*_*k*_.

(b) If *α*_*i*_=*γ* + *ρ*_*k*_, the *k*th reaction term appears as a limit in the limiting equation. The limit of the *k*th reaction term is discrete if *α*_*i*_=0, while it is a continuous variable with the limit of its propensity if *α*_*i*_>0.

(c) There is no *k* satisfying *α*_*i*_<*γ* + *ρ*_*k*_in the equation for species *S*_*i*_with *γ*=*γ*_*i*_due to the definition of *γ*_*i*_given in Step 9.

12. In the limiting equation for each species *S*_*i*_with *γ*_*i*_=*γ*, we approximate propensities in the reaction terms. Suppose that the normalized species number for *S*_*j*_appears in the propensities. 

(a) If *γ*_*j*_>*γ*, the limit of the normalized species number for *S*_*j*_is its initial value.

(b) If *γ*_*j*_=*γ*, the limit of the normalized species number for *S*_*j*_appears as a variable in the propensities in the limiting equation.

(c) If *γ*_*j*_<*γ*, the limit of the normalized species number for *S*_*j*_is expressed as a function of the limits of the normalized species numbers for *S*_*i*_with *γ*_*i*_=*γ*. The function for *S*_*j*_is obtained by dividing the equation for *S*_*j*_by $N^{\underset{k\in \Gamma_{j}^{+}\cup \Gamma_{j}^{-}}{\max}(\gamma +\rho_{k})-\alpha_{j}}$ and letting *N* go to infinity.

13. If a limiting model is not closed, consider limiting equations for some linear combinations of species selected in Step 5 whose natural time scale exponents are equal to the chosen *γ*.

The method for multiscale approximation described above can be applied to general chemical reaction networks containing different scales in species numbers and reaction rate constants. We can apply the method in case the rates of chemical reactions are determined by law of mass action and when there is no species whose number is either zero or infinity at all times. As given in [9], in the reaction network involving *∅*→*S*_1_, *∅*→*S*_2_, *∅*→*S*_3_, *S*_1_ + *S*_2_→*∅*, and *S*_1_ + *S*_3_→*∅*, convergence of the limit for the scaled species numbers may not be guaranteed at some time scales. Suppose that production rate of *S*_1_ is larger than that of *S*_2_but with the same order of magnitude, and that production rate of *S*_3_ is much smaller than those of *S*_1_and *S*_2_. Then, *X*_1_(*t*) may blow up to infinity and *X*_2_(*t*) may go to zero at some time scales. In this case, the method is not applicable.

## Results and discussion

### Model description

We analyze a heat shock response model of *E. coli* developed by Srivastava, Peterson, and Bentley [11]. The heat shock response model gives a simplified mechanism occurring in the *E. coli* to respond to high temperature. Heat causes unfolding, misfolding, or aggregation of proteins, and cells overcome the heat stress by producing heat shock proteins, which refold or degrade denatured proteins. In *E. coli*, *σ*^32^factors play an important role in recovery from the stress under the high temperature. *σ*^32^factors catalyze production of the heat shock proteins such as chaperon proteins and other proteases. In this model, *J* denotes a chaperon complex, *FtsH* represents a *σ*^32^-regulated stress protein, and *GroEL* is a *σ*^32^-mediated stress response protein.

*σ*^32^ factors are in three different forms, free *σ*^32^protein, *σ*^32^ combined with RNA polymerase (*Eσ*^32^), and *σ*^32^ combined with a chaperon complex (*σ*^32^-*J*). Under the normal situation without stress, most of the *σ*^32^ factors combine with chaperon complexes and form *σ*^32^-*J*. A chaperon complex *J* keeps *σ*^32^factors in an inactive form, and *σ*^32^factors can directly respond to the stress by changing into different forms. When there exist *σ*^32^factors combined with chaperon complexes, *FtsH* catalyzes degradation of *σ*^32^ factors. Thus, if enough *σ*^32^-regulated stress proteins are produced, *σ*^32^factors are degraded.

Not only *σ*^32^factors, but recombinant proteins also require chaperon complexes to form a complex so that denatured protein can be fixed. Therefore, *σ*^32^factors and recombinant proteins compete to bind chaperon complexes, and different levels of binding affinity of recombinant proteins to chaperon complexes change the evolution of the system state. In the model, we assume that *σ*^32^ factors and recombinant proteins have the same affinity to bind to chaperon complexes. The system is sensitive to the amount and forms of *σ*^32^ factors: a small decrease of *σ*^32^factors causes a large reduction of production of chaperon complexes and *σ*^32^-regulated stress proteins, and the ratio of three different forms of *σ*^32^factors determines system dynamics in the stress response [11]. The total initial number of molecules of *σ*^32^ factors in each cell is small [11] (also see initial values for *S*_2_, *S*_3_, and *S*_7_ which are 1, 1, and 7 in Table 1), and the stochastic model is appropriate to be considered.

**Table 1:** Species in the heat shock response model of E. coli and their initial values

						
*X*_1_	=	# of *S*_1_	*σ*^32^ mRNA	*X*_1_(0)	=	10
*X*_2_	=	# of *S*_2_	*σ*^32^ protein	*X*_2_(0)	=	1
*X*_3_	=	# of *S*_3_	*Eσ*^32^	*X*_3_(0)	=	1
*X*_4_	=	# of *S*_4_	*FtsH*	*X*_4_(0)	=	93
*X*_5_	=	# of *S*_5_	*GroEL*	*X*_5_(0)	=	172
*X*_6_	=	# of *S*_6_	*J*	*X*_6_(0)	=	54
*X*_7_	=	# of *S*_7_	*σ*^32^-*J*	*X*_7_(0)	=	7
*X*_8_	=	# of *S*_8_	Recombinant protein	*X*_8_(0)	=	50
*X*_9_	=	# of *S*_9_	*J*-Recombinant protein	*X*_9_(0)	=	0

The model involves 9 species and 18 reactions. Denote *s*_0_ as the number of species and *r*_0_ as the number of reactions. Let *X*(*t*) be a state vector whose *i*th component represents the number of molecules of species *S*_*i*_ at time *t* for *i*=1,⋯,*s*_0_. Define a random process which counts the number of times that the *k*th reaction occurs by time *t* as

$$R_{k}^{t}\left(\lambda_{k}(X)\right)\;\;\equiv \;\;Y_{k}\left(\overset{t}{\underset{0}{\int }}\lambda_{k}(X(s))\;\mathrm{ds}\right),\;k=1,\cdots \;,r_{0},$$

where *λ*_*k*_(*X*) is the propensity of the *k*th reaction and the *Y*_*k*_’s are independent unit Poisson processes. Therefore, $R_{k}^{t}(\cdot )$ is a nonnegative integer-valued random process increasing by 1. As *λ*_*k*_(·) gets large, the moment when $R_{k}^{t}(\lambda_{k}(\cdot ))$ increases becomes more frequent. Let *ν*_*ik*_($\nu_{\mathrm{ik}}^{\prime }$) be the number of molecules of *S*_*i*_ that are consumed (produced) in the *k*th reaction. Define *ν*_*k*_($\nu_{k}^{\prime }$) as an *s*_0_-dimensional vector whose *i*th component is *ν*_*ik*_($\nu_{\mathrm{ik}}^{\prime }$). Then, *X*(*t*) is given as

(1) $$X(t)\;\;=\;\;X(0)+\overset{r_{0}}{\underset{k=1}{\sum }}R_{k}^{t}\left(\lambda_{k}(X)\right)(\nu_{k}^{\prime }-\nu_{k}).$$

That is, species numbers at time *t* are expressed in terms of their initial values and sum of the number of times that each reaction occurs multiplied by net molecule changes in the corresponding reaction. In our model, the system of equations are derived using a set of reactions in Table 2 as:

(2) $$\;\begin{array}{l} X_{1}(t)=X_{1}(0)+R_{13}^{t}(\kappa_{13}^{\prime })-R_{14}^{t}(\kappa_{14}^{\prime }X_{1}),\\ X_{2}(t)=X_{2}(0)+R_{3}^{t}(\kappa_{3}^{\prime }X_{3})+R_{4}^{t}(\kappa_{4}^{\prime }X_{1})+R_{5}^{t}(\kappa_{5}^{\prime }X_{3})\\ \;+R_{6}^{t}(\kappa_{6}^{\prime }X_{3})+R_{7}^{t}(\kappa_{7}^{\prime }X_{3})+R_{8}^{t}(\kappa_{8}^{\prime }X_{7})-R_{2}^{t}(\kappa_{2}^{\prime }X_{2})\\ \;-R_{9}^{t}(\kappa_{9}^{\prime }X_{2}X_{6}),\\ X_{3}(t)=X_{3}(0)+R_{2}^{t}(\kappa_{2}^{\prime }X_{2})-R_{3}^{t}(\kappa_{3}^{\prime }X_{3})-R_{5}^{t}(\kappa_{5}^{\prime }X_{3})\\ \;-R_{6}^{t}(\kappa_{6}^{\prime }X_{3})-R_{7}^{t}(\kappa_{7}^{\prime }X_{3}),\\ X_{4}(t)=X_{4}(0)+R_{6}^{t}(\kappa_{6}^{\prime }X_{3})-R_{18}^{t}(\kappa_{18}^{\prime }X_{4}),\\ X_{5}(t)=X_{5}(0)+R_{5}^{t}(\kappa_{5}^{\prime }X_{3})-R_{16}^{t}(\kappa_{16}^{\prime }X_{5}),\\ X_{6}(t)=X_{6}(0)+R_{7}^{t}(\kappa_{7}^{\prime }X_{3})+R_{8}^{t}(\kappa_{8}^{\prime }X_{7})+R_{12}^{t}(\kappa_{12}^{\prime }X_{9})\\ \;+R_{15}^{t}(\kappa_{15}^{\prime }X_{4}X_{7})-R_{9}^{t}(\kappa_{9}^{\prime }X_{2}X_{6})-R_{10}^{t}(\kappa_{10}^{\prime }X_{6}X_{8})\\ \;-R_{17}^{t}(\kappa_{17}^{\prime }X_{6}),\\ X_{7}(t)=X_{7}(0)+R_{9}^{t}(\kappa_{9}^{\prime }X_{2}X_{6})-R_{8}^{t}(\kappa_{8}^{\prime }X_{7})-R_{15}^{t}(\kappa_{15}^{\prime }X_{4}X_{7}),\\ X_{8}(t)=X_{8}(0)+R_{1}^{t}(\kappa_{1}^{\prime })+R_{12}^{t}(\kappa_{12}^{\prime }X_{9})-R_{10}^{t}(\kappa_{10}^{\prime }X_{6}X_{8})\\ \;-R_{11}^{t}(\kappa_{11}^{\prime }X_{8}),\\ X_{9}(t)=X_{9}(0)+R_{10}^{t}(\kappa_{10}^{\prime }X_{6}X_{8})-R_{12}^{t}(\kappa_{12}^{\prime }X_{9}). \end{array}$$

**Table 2:** Reactions in the heat shock response model of E. coli

	**Reaction**	**Transition**
R1	$\emptyset \overset{\text{gene}}{\to }S_{8}$	Recombinant protein synthesis
R2	*S*_2_→*S*_3_	Holoenzyme association
R3	*S*_3_→*S*_2_	Holoenzyme disassociation
R4	$\emptyset \overset{S_{1}}{\to }S_{2}$	*σ*^32^ translation
R5	$S_{3}\overset{\text{gene}}{\to }S_{2}+S_{5}$	*GroEL* synthesis
R6	$S_{3}\overset{\text{gene}}{\to }S_{2}+S_{4}$	*FtsH* synthesis
R7	$S_{3}\overset{\text{gene}}{\to }S_{2}+S_{6}$	*J*-production
R8	*S*_7_→*S*_2_ + *S*_6_	*σ*^32^-*J*-disassociation
R9	*S*_2_ + *S*_6_→*S*_7_	*σ*^32^-*J*-association
R10	*S*_6_ + *S*_8_→*S*_9_	Recombinant protein-*J* association
R11	*S*_8_→∅	Recombinant protein degradation
R12	*S*_9_→*S*_6_ + *S*_8_	Recombinant protein-*J* disassociation
R13	$\emptyset \overset{\text{gene}}{\to }S_{1}$	*σ*^32^ transcription
R14	*S*_1_→∅	*σ*^32^ mRNA decay
R15	$S_{7}\overset{S_{4}}{\to }S_{6}$	*σ*^32^ degradation
R16	$S_{5}\to \emptyset$	*GroEL* degradation
R17	$S_{6}\to \emptyset$	*J*-disassociation
R18	$S_{4}\to \emptyset$	*FtsH* degradation

In Reaction 5, 6, and 7, we assume that the number of molecules of each gene is 1 and that these reactions are effectively unimolecular. Similarly, Reactions 1 and 13 are treated as production from a source.

$\kappa_{k}^{\prime }$ represents the stochastic reaction rate constant for the *k*th reaction, and their values from [11] are given in Table 3.

**Table 3:** Stochastic reaction rate constants in the heat shock response model of E. coli

**Rates**		**Rates**	
$\kappa_{1}^{\prime }$	4.00×10^0^	$\kappa_{10}^{\prime }$	3.62×10^−4^
$\kappa_{2}^{\prime }$	7.00×10^−1^	$\kappa_{11}^{\prime }$	9.99×10^−5^
$\kappa_{3}^{\prime }$	1.30×10^−1^	$\kappa_{12}^{\prime }$	4.40×10^−5^
$\kappa_{4}^{\prime }$	7.00×10^−3^	$\kappa_{13}^{\prime }$	1.40×10^−5^
$\kappa_{5}^{\prime }$	6.30×10^−3^	$\kappa_{14}^{\prime }$	1.40×10^−6^
$\kappa_{6}^{\prime }$	4.88×10^−3^	$\kappa_{15}^{\prime }$	1.42×10^−6^
$\kappa_{7}^{\prime }$	4.88×10^−3^	$\kappa_{16}^{\prime }$	1.80×10^−8^
$\kappa_{8}^{\prime }$	4.40×10^−4^	$\kappa_{17}^{\prime }$	6.40×10^−10^
$\kappa_{9}^{\prime }$	3.62×10^−4^	$\kappa_{18}^{\prime }$	7.40×10^−11^

We convert deterministic rate constants in [11] using the volume of *E. coli* which is assumed to be 1.5×10^−15^*L*.

We derive the limiting models in three time scales, which approximate a full network in a certain time period involving a subset of species and reactions. In what follows, $Z_{i}^{\gamma }$ is a limit of the scaled species number of *S*_*i*_ at some time scales depending on *γ*, and as *γ* gets larger the times are in the later stage. Note that the exponent *γ*in $Z_{i}^{\gamma }$ does not imply (*Z*_*i*_)^*γ*^ but it shows dependence of $Z_{i}^{\gamma }$ on *γ*. Let *κ*_*k*_ be a scaled reaction rate constant for the *k*th reaction. In the first time scale (when the times are in the early stage), the subnetwork governed by

(3) $$\begin{array}{l} Z_{2}^{0}(t)=Z_{2}^{0}(0)+R_{3}^{t}(\kappa_{3}Z_{3}^{0})+R_{4}^{t}(\kappa_{4}Z_{1}^{0}(0))-R_{2}^{t}(\kappa_{2}Z_{2}^{0}),\\ Z_{3}^{0}(t)=Z_{3}^{0}(0)+R_{2}^{t}(\kappa_{2}Z_{2}^{0})-R_{3}^{t}(\kappa_{3}Z_{3}^{0}),\\ Z_{8}^{0}(t)=Z_{8}^{0}(0)+R_{1}^{t}(\kappa_{1}), \end{array}$$

approximates the network when the times are of order 1 sec. Denote $Z_{23}^{1}$ as the limit of the addition of the scaled species numbers for *S*_2_and *S*_3_. In the second time scale (when the times are in the medium stage), the subnetwork governed by

(4) $$\;\begin{array}{l} Z_{23}^{1}(t)=Z_{23}^{1}(0)+R_{4}^{t}(\kappa_{4}Z_{1}^{1}(0)),\\ Z_{6}^{1}(t)=Z_{6}^{1}(0)+R_{7}^{t}\left(\frac{\kappa_{2}\kappa_{7}}{\kappa_{2}+\kappa_{3}}Z_{23}^{1}\right)+R_{12}^{t}(\kappa_{12}Z_{9}^{1}(0))\\ \;+R_{15}^{t}(\kappa_{15}Z_{4}^{1}(0)Z_{7}^{1})-R_{10}^{t}(\kappa_{10}Z_{6}^{1}Z_{8}^{1}),\\ Z_{7}^{1}(t)=Z_{7}^{1}(0)-R_{15}^{t}(\kappa_{15}Z_{4}^{1}(0)Z_{7}^{1}),\\ Z_{8}^{1}(t)=Z_{8}^{1}(0)+\kappa_{1}t, \end{array}$$

approximates the network at the times of order 100 sec. In the third time scale, set the limit of the averaged scaled species numbers of fast-fluctuating species *S*_2_, *S*_3_, and *S*_6_ as

$$\begin{array}{l} {\bar{Z}}_{2}^{2}(t)\equiv \frac{\kappa_{3}}{\kappa_{2}+\kappa_{3}}Z_{23}^{2}(t),\\ {\bar{Z}}_{3}^{2}(t)\equiv \frac{\kappa_{2}}{\kappa_{2}+\kappa_{3}}Z_{23}^{2}(t),\\ {\bar{Z}}_{6}^{2}(t)\equiv \frac{\kappa_{7}{\bar{Z}}_{3}^{2}(s)+\kappa_{12}Z_{9}^{2}(s)}{\kappa_{10}Z_{8}^{2}(s)}. \end{array}$$

When the times are in a late stage, the subnetwork governed by

(5) $$\;\begin{array}{l} Z_{1}^{2}(t)=Z_{1}^{2}(0)+R_{13}^{t}(\kappa_{13})-R_{14}^{t}(\kappa_{14}Z_{1}^{2}),\\ Z_{23}^{2}(t)=Z_{23}^{2}(0)+\int_{0}^{t}\left[\kappa_{4}Z_{1}^{2}(s)-\frac{\kappa_{3}\kappa_{9}}{\kappa_{2}+\kappa_{3}}Z_{23}^{2}(s)\right)\\ \;\times \left(\left(\frac{\frac{\kappa_{2}\kappa_{7}}{\kappa_{2}+\kappa_{3}}Z_{23}^{2}(s)+\kappa_{12}Z_{9}^{2}(s)}{\kappa_{10}Z_{8}^{2}(s)}\right)\right]\;\mathrm{ds}\\ \;\equiv Z_{23}^{2}(0)+\int_{0}^{t}\left[\kappa_{4}Z_{1}^{2}(s)-\kappa_{9}{\bar{Z}}_{2}^{2}(s){\bar{Z}}_{6}^{2}(s)\right]\;\mathrm{ds},\\ Z_{4}^{2}(t)=Z_{4}^{2}(0)+\int_{0}^{t}\left(\frac{\kappa_{2}\kappa_{6}}{\kappa_{2}+\kappa_{3}}Z_{23}^{2}(s)-\kappa_{18}Z_{4}^{2}(s)\right)\;\mathrm{ds}\\ \;\equiv Z_{4}^{2}(0)+\int_{0}^{t}\left(\kappa_{6}{\bar{Z}}_{3}^{2}(s)-\kappa_{18}Z_{4}^{2}(s)\right)\;\mathrm{ds},\\ Z_{5}^{2}(t)=Z_{5}^{2}(0)+\int_{0}^{t}\left(\frac{\kappa_{2}\kappa_{5}}{\kappa_{2}+\kappa_{3}}Z_{23}^{2}(s)-\kappa_{16}Z_{5}^{2}(s)\right)\;\mathrm{ds}\\ \;\equiv Z_{5}^{2}(0)+\int_{0}^{t}\left(\kappa_{5}{\bar{Z}}_{3}^{2}(s)-\kappa_{16}Z_{5}^{2}(s)\right)\;\mathrm{ds},\\ Z_{8}^{2}(t)=Z_{8}^{2}(0)+\int_{0}^{t}\left(\;\kappa_{1}-\frac{\kappa_{2}\kappa_{7}}{\kappa_{2}+\kappa_{3}}Z_{23}^{2}(s)-\kappa_{11}Z_{8}^{2}(s)\;\right)\mathrm{ds}\\ \;\equiv Z_{8}^{2}(0)+\int_{0}^{t}\left(\kappa_{1}-\kappa_{7}{\bar{Z}}_{3}^{2}(s)-\kappa_{11}Z_{8}^{2}(s)\right)\;\mathrm{ds},\\ Z_{9}^{2}(t)=Z_{9}^{2}(0)+\int_{0}^{t}\frac{\kappa_{2}\kappa_{7}}{\kappa_{2}+\kappa_{3}}Z_{23}^{2}(s)\;\mathrm{ds}\\ \;\equiv Z_{9}^{2}(0)+\int_{0}^{t}\kappa_{7}{\bar{Z}}_{3}^{2}(s)\;\mathrm{ds}, \end{array}$$

approximates the network at the times of order 10,000 sec. Detailed derivation is given in the later sections. Note that it is possible to identify different numbers of time scales depending on the scaling of the species numbers and reaction rate constants. In the heat shock response model of *E. coli*, it is possible to obtain approximate models with two or four time scales. However, if the number of time scales are too many, the limiting model in each time scale may involve one species and a few number of reactions and the model in this case may not be interesting to consider.

### Derivation of the scaled models

The stochastic equations given in Equations (2) describe temporal evolution of the species numbers. For example, the equations for species *S*_2_and *S*_3_ are

(6a) $$\;\begin{array}{l} X_{2}(t)=X_{2}(0)+R_{3}^{t}\left(\kappa_{3}^{\prime }X_{3}\right)+R_{4}^{t}\left(\kappa_{4}^{\prime }X_{1}\right)+R_{5}^{t}\left(\kappa_{5}^{\prime }X_{3}\right)\\ \;+R_{6}^{t}\left(\kappa_{6}^{\prime }X_{3}\right)+R_{7}^{t}\left(\kappa_{7}^{\prime }X_{3}\right)+R_{8}^{t}\left(\kappa_{8}^{\prime }X_{7}\right)\;-\;R_{2}^{t}\left(\kappa_{2}^{\prime }X_{2}\right)\\ \;-R_{9}^{t}\left(\kappa_{9}^{\prime }X_{2}X_{6}\right), \end{array}$$

(6b) $$\;\begin{array}{l} X_{3}(t)=X_{3}(0)+R_{2}^{t}\left(\kappa_{2}^{\prime }X_{2}\right)-R_{3}^{t}\left(\kappa_{3}^{\prime }X_{3}\right)-R_{5}^{t}\left(\kappa_{5}^{\prime }X_{3}\right)\\ \;-R_{6}^{t}\left(\kappa_{6}^{\prime }X_{3}\right)-R_{7}^{t}\left(\kappa_{7}^{\prime }X_{3}\right). \end{array}$$

In Equation (6), species numbers of *S*_2_and *S*_3_ are determined by the times when reactions occur and by the number of times that reactions happen. On the other hand, reaction time and frequency are determined by propensities which are some functions of species numbers. Therefore, reaction rates and species numbers interact one another. Reaction rates vary from *O*(10^−11^) to *O*(1) as we see in Table 3, and species numbers in this model are from *O*(1) to *O*(10^4^) as we see later in the simulation of the full network. We express each species number and rate constant in terms of powers of a common number with different weights on exponents. Define *N*_0_=100 as a fixed unitless constant used to express the magnitude of the species numbers and the reaction rate constants. Define *α*_*i*_ for *i*=1,⋯,*s*_0_ and *β*_*k*_ for *k*=1,⋯,*r*_0_ as the scaling exponent for species *S*_*i*_ and for the reaction rate constant $\kappa_{k}^{\prime }$. We express the reaction rate constants in a form of $N_{0}^{\beta_{k}}\kappa_{k}$ where *κ*_*k*_ is of order 1 and is determined so that $\kappa_{k}^{\prime }=N_{0}^{\beta_{k}}\kappa_{k}$. For example, we have $\kappa_{6}^{\prime }=4.88\times 10^{-3}$ and we can choose *β*_6_=−1 so that the reaction rate is expressed as $\kappa_{6}^{\prime }=0.488\times N_{0}^{\beta_{6}}$. Assuming that *N*_0_ is large, we replace *N*_0_ by *N* and express the process as $X_{i}^{N}(t)$ to show dependence of the species numbers on *N*. Note that {*X*^*N*^(*t*)} is a family of processes depending on *N* and $X_{i}^{N}(t)=X_{i}(t)$ when *N*=*N*_0_. Then, the equation for $X_{3}^{N}$ is given as

$$\;\begin{array}{l} X_{3}^{N}(t)=X_{3}^{N}(0)+R_{2}^{t}\left(N^{\beta_{2}}\kappa_{2}X_{2}^{N}\right)-R_{3}^{t}\left(N^{\beta_{3}}\kappa_{3}X_{3}^{N}\right)\\ \;-R_{5}^{t}\;\left(\;N^{\beta_{5}}\kappa_{5}X_{3}^{N}\;\right)\;-\;R_{6}^{t}\;\left(\;N^{\beta_{6}}\kappa_{6}X_{3}^{N}\;\right)\;-\;R_{7}^{t}\;\left(N^{\beta_{7}}\kappa_{7}X_{3}^{N}\right)\;, \end{array}$$

where $X_{3}^{N}(0)$ is defined later so that $X_{3}^{N}(0)=X_{3}(0)$ when *N*=*N*_0_. Since the numbers of molecules of species are in different orders of magnitude, we scale the number of molecules of the *i*th species by *N*^*α*^_*i*_ and set a normalized species number as

$$Z_{i}^{N}(t)\;\;=\;\;N^{-\alpha_{i}}X_{i}^{N}(t).$$

The *i*th species number may have different orders of magnitude at different times so *α*_*i*_ may have different values for different time scales. Now, we set the initial values as

(7) $$X_{i}^{N}(0)\;\;\equiv \;\;\left\lfloor {\left(\frac{N}{N_{0}}\right)}^{\alpha_{i}}X_{i}(0)\right\rfloor ,$$

so that $X_{i}^{N_{0}}(0)=X_{i}(0)$ and $\underset{N\to \infty }{\lim}Z_{i}^{N}(0)=\underset{N\to \infty }{\lim}$$N^{-\alpha_{i}}X_{i}^{N}(0)=N_{0}^{-\alpha_{i}}X_{i}(0)$.

Next, we scale the propensities of reactions by replacing $X_{i}^{N}$ by $N^{\alpha_{i}}Z_{i}^{N}$ and replacing $\kappa_{k}^{\prime }$ by *N*^*β*^_*k*_*κ*_*k*_. For example, consider the 9th reaction term in (6a). Replacing $\kappa_{9}^{\prime }$ by *N*^*β*^_9_*κ*_9_, $X_{2}^{N}$ by $N^{\alpha_{2}}Z_{2}^{N}$, and $X_{6}^{N}$ by $N^{\alpha_{6}}Z_{6}^{N}$, the 9th reaction term becomes

(8) $$R_{9}^{t}(\kappa_{9}^{\prime }X_{2}^{N}X_{6}^{N})=R_{9}^{t}(N^{\beta_{9}+\alpha_{2}+\alpha_{6}}\kappa_{9}Z_{2}^{N}Z_{6}^{N}).$$

For simplicity, set *ρ*_9_=*β*_9_ + *α*_2_ + *α*_6_ and define a scaling exponent in the propensity of the *k*th reaction term as

$$\rho_{k}\equiv \beta_{k}+\nu_{k}\cdot \alpha ,$$

where $\alpha ={\left(\alpha_{1},\cdots \;,\alpha_{s_{0}}\right)}^{T}$ and $\nu_{k}={\left(\nu_{1k},\cdots \;,\nu_{s_{0}k}\right)}^{T}$. Here, *ν*_*ik*_ gives the number of molecules of species *S*_*i*_consumed in the *k*th reaction. Then, (8) is rewritten as

$$R_{9}^{t}\left(\kappa_{9}^{\prime }X_{2}^{N}X_{6}^{N}\right)=R_{9}^{t}\left(N^{\rho_{9}}\kappa_{9}Z_{2}^{N}Z_{6}^{N}\right).$$

Dividing (6a) by *N*^*α*^_2_ and (6b) by *N*^*α*^_3_ and scaling the propensities, we get

(9a) $$\;\begin{array}{l} Z_{2}^{N}(t)=Z_{2}^{N}(0)+N^{-\alpha_{2}}\left[R_{3}^{t}\left(N^{\rho_{3}}\kappa_{3}Z_{3}^{N}\right)\;+\;R_{4}^{t}\left(N^{\rho_{4}}\kappa_{4}Z_{1}^{N}\right)\right)\\ \;+R_{5}^{t}\left(N^{\rho_{5}}\kappa_{5}Z_{3}^{N}\right)+R_{6}^{t}\left(N^{\rho_{6}}\kappa_{6}Z_{3}^{N}\right)\\ \;+R_{7}^{t}\left(N^{\rho_{7}}\kappa_{7}Z_{3}^{N}\right)+R_{8}^{t}\left(N^{\rho_{8}}\kappa_{8}Z_{7}^{N}\right)\\ \;\left(-R_{2}^{t}\left(N^{\rho_{2}}\kappa_{2}Z_{2}^{N}\right)-R_{9}^{t}\left(N^{\rho_{9}}\kappa_{9}Z_{2}^{N}Z_{6}^{N}\right)\right], \end{array}$$

(9b) $$\;\begin{array}{l} Z_{3}^{N}(t)=Z_{3}^{N}(0)+N^{-\alpha_{3}}\left[R_{2}^{t}\left(N^{\rho_{2}}\kappa_{2}Z_{2}^{N}\right)\;-\;R_{3}^{t}\left(N^{\rho_{3}}\kappa_{3}Z_{3}^{N}\right)\right)\\ \;-R_{5}^{t}\left(N^{\rho_{5}}\kappa_{5}Z_{3}^{N}\right)-R_{6}^{t}\left(N^{\rho_{6}}\kappa_{6}Z_{3}^{N}\right)\\ \;\left(-R_{7}^{t}\left(N^{\rho_{7}}\kappa_{7}Z_{3}^{N}\right)\right]. \end{array}$$

For each reaction, *ρ*_*k*_is given in terms of *α*_*i*_and *β*_*k*_ in the Additional file 1: Table S1.

We are interested in dynamics of species numbers $Z_{2}^{N}(t)$ and $Z_{3}^{N}(t)$ in various stages of time period. In the early stage of time period, normalized species numbers of *S*_2_ and *S*_3_ are very close to their scaled initial values, since these species numbers have not changed yet. In the medium stage of time period, the normalized species numbers of *S*_2_and *S*_3_ are asymptotically equal to non-constant limits. In the late stage of time period, the normalized species numbers of *S*_2_ and *S*_3_fluctuate very rapidly and their averaged behavior is captured in terms of some function of other species numbers.

We want to express the time scale of each species in terms of power of *N*. First, we express order of magnitude of a specific time period of interest as a power of *N* with a time scale exponent *γ*. Applying a time change by replacing *t* by *N*^*γ*^*t*in $Z_{i}^{N}(t)$, we define a variable for the normalized species numbers after a time change as

(10) $$Z_{i}^{N,\gamma }(t)\;\equiv \;N^{-\alpha_{i}}X_{i}^{N}(tN^{\gamma })=Z_{i}^{N}(tN^{\gamma }).$$

Then, $Z_{i}^{N,\gamma }(t)$ gives a normalized species number at the times of order *N*^*γ*^. A *natural time scale* of *S*_*i*_is the time when $Z_{i}^{N,\gamma }(t)$ has a nonzero finite limit which is not constant and of order 1.

Changing a time variable by replacing *t* by *N*^*γ*^*t* in (9a) and (9b), the normalized species numbers of *S*_2_and *S*_3_after a time change satisfy

(11a) $$\;\begin{array}{l} Z_{2}^{N,\gamma }(t)=Z_{2}^{N,\gamma }(0)+N^{-\alpha_{2}}\left[R_{3}^{t}\left(N^{\gamma +\rho_{3}}\kappa_{3}Z_{3}^{N,\gamma }\right)\right)\\ \;+R_{4}^{t}\left(N^{\gamma +\rho_{4}}\kappa_{4}Z_{1}^{N,\gamma }\right)+R_{5}^{t}\left(N^{\gamma +\rho_{5}}\kappa_{5}Z_{3}^{N,\gamma }\right)\\ \;+R_{6}^{t}\left(N^{\gamma +\rho_{6}}\kappa_{6}Z_{3}^{N,\gamma }\right)+R_{7}^{t}\left(N^{\gamma +\rho_{7}}\kappa_{7}Z_{3}^{N,\gamma }\right)\\ \;+R_{8}^{t}\left(N^{\gamma +\rho_{8}}\kappa_{8}Z_{7}^{N,\gamma }\right)-R_{2}^{t}\left(N^{\gamma +\rho_{2}}\kappa_{2}Z_{2}^{N,\gamma }\right)\\ \;-\left(R_{9}^{t}\left(N^{\gamma +\rho_{9}}\kappa_{9}Z_{2}^{N,\gamma }Z_{6}^{N,\gamma }\right)\right], \end{array}$$

(11b) $$\;\begin{array}{l} Z_{3}^{N,\gamma }(t)=Z_{3}^{N,\gamma }(0)+N^{-\alpha_{3}}\left[R_{2}^{t}\left(N^{\gamma +\rho_{2}}\kappa_{2}Z_{2}^{N,\gamma }\right)\right)\\ \;-R_{3}^{t}\left(N^{\gamma +\rho_{3}}\kappa_{3}Z_{3}^{N,\gamma }\right)-R_{5}^{t}\left(N^{\gamma +\rho_{5}}\kappa_{5}Z_{3}^{N,\gamma }\right)\\ \;\left(-R_{6}^{t}\left(N^{\gamma +\rho_{6}}\kappa_{6}Z_{3}^{N,\gamma }\right)-R_{7}^{t}\left(N^{\gamma +\rho_{7}}\kappa_{7}Z_{3}^{N,\gamma }\right)\right], \end{array}$$

where *N*^*γ*^in each propensity comes from the change of the time variable. Here, the initial values may depend on *γ*, since we can choose different values for *α*_*i*_for each *γ*due to changes in order of magnitude of species numbers in time. The stochastic equations after scaling and a time change for all species are given in the Additional file 1: Section 1.

### Balance conditions

Our goal is to approximate dynamics of the full network in the heat shock response model of *E. coli* in specific times of interest in terms of simplified subnetworks preserving significant biological features. In each time period of interest, we obtain a nondegenerate limiting model which is not equal to zero and does not blow up to infinity. In this section, we introduce balance conditions which help us to choose appropriate values for the scaling exponents *α*_*i*_’s and *β*_*k*_’s so that the limit is nonzero finite. For each time period of interest of order $N_{0}^{\gamma }$ where *N*_0_=100, we choose values for scaling exponents so that orders of magnitude of the species number for *S*_*i*_ and the *k*th reaction rate constant are about $N_{0}^{\alpha_{i}}$ and $N_{0}^{\beta_{k}}$, respectively. That is,

$$\begin{array}{ll} Z_{i}^{N_{0},\gamma }(t)=\frac{X_{i}^{N_{0}}(tN_{0}^{\gamma })}{N_{0}^{\alpha_{i}}}=O(1),\\ \;\kappa_{k}=\frac{\kappa_{k}^{\prime }}{N_{0}^{\beta_{k}}}=O(1).\; & \; \end{array}$$

It is natural to choose *β*_*k*_’s in monotone decreasing manner in *k*, since $\kappa_{k}^{\prime }$’s are in monotone decreasing order as shown in Table 3. In Table 3, the production rates from a source are the rates per second. The unimolecular reaction rates are the rates per molecule per second while the bimolecular reaction rates are the rates per a pair of molecules per second. Since the reaction rates are expressed in different units, we separate rate constants into three classes based on the number of reactants and assume that monotonicity of *β*_*k*_’s holds in each class of reactions. In other words, we choose *β*_*k*_’s so that

$$\begin{array}{lll} \beta_{1} & \geq \beta_{13},\; & \;\\ \beta_{2} & \geq \beta_{3}\geq \beta_{4}\geq \beta_{5}\geq \beta_{6}\geq \beta_{7}\geq \beta_{8}\geq \beta_{11}\;\geq \;\beta_{12}\;\geq \;\beta_{14}\; & \;\\ & \geq \beta_{16}\geq \beta_{17}\geq \beta_{18},\;\text{and}\; & \;\\ \beta_{9} & \geq \beta_{10}\geq \beta_{15}.\; & \; \end{array}$$

Next, in order to make the normalized specie number $Z_{i}^{N,\gamma }(t)$ balanced, it is required that the rates of production and consumption of *S*_*i*_should be in the same order of magnitude. If the order of magnitude of production rate is larger than that of consumption, the normalized species number asymptotically goes to infinity. In the opposite case, the normalized species number asymptotically becomes zero. Therefore, for each species *S*_*i*_, we set the balance equation for *α*_*i*_’s and *β*_*k*_’s so that the maximal exponent in the propensities of the reactions producing *S*_*i*_ is equal to that in the propensities of the reactions consuming *S*_*i*_. For example, to obtain a balance equation for species *S*_2_, we compare the scaling exponents in propensities of reactions involving *S*_2_using (11a), and set the maximal exponents of production and consumption of *S*_2_ equal. Similarly, using (11b), we set the maximal exponents in the production rates and the consumption rates of *S*_3_ equal. Then, the balance equations for species *S*_2_and *S*_3_ are

(12a) $$\max(\rho_{3},\rho_{4},\rho_{5},\rho_{6},\rho_{7},\rho_{8})=\max(\rho_{2},\rho_{9}),$$

(12b) $$\rho_{2}=\max(\rho_{3},\rho_{5},\rho_{6},\rho_{7}).$$

If the maximal orders of magnitudes of production and consumption rates for *S*_2_ are different from each other, the species number of *S*_2_should be large enough so that a difference between production and consumption of *S*_*i*_ is not noticeable. In other words if *α*_*i*_’s and *β*_*k*_’s do not satisfy (12a), *α*_2_should be at least as large as the scaling exponents located in all reaction terms in (11a) to prevent the limit becoming zero or blowing up to infinity. Similarly, in case (12b) is not satisfied, *α*_3_ should be at least as large as the scaling exponents located in the reaction terms in (11b) to prevent the limit becoming zero or blowing up to infinity.

(13) $$\begin{array}{l} \alpha_{2}\geq \gamma +\max(\rho_{2},\rho_{3},\rho_{4},\rho_{5},\rho_{6},\rho_{7},\rho_{8},\rho_{9}),\\ \alpha_{3}\geq \gamma +\max(\rho_{2},\rho_{3},\rho_{5},\rho_{6},\rho_{7}), \end{array}$$

Solving (13) for *γ*, we get the following time-scale constraints:

(14a) $$\gamma \leq \alpha_{2}-\max(\rho_{2},\rho_{3},\rho_{4},\rho_{5},\rho_{6},\rho_{7},\rho_{8},\rho_{9})\equiv u_{2},$$

(14b) $$\gamma \leq \alpha_{3}-\max(\rho_{2},\rho_{3},\rho_{5},\rho_{6},\rho_{7})\equiv u_{3}.$$

Inequalities in (14) mean that if maximal production and consumption rates are not balanced either for *S*_2_ or *S*_3_, the chosen set of values for scaling exponents can be used to approximate the dynamics of the full network up to times of order *N*^*u*^_2_ or *N*^*u*^_3_. For times later than those of order *N*^*u*^_2_or *N*^*u*^_3_, we need to choose another set of values for scaling exponents based on the balance equations. We call the balance equation and the time-scale constraint for each species as the *species balance condition*. If either (12a) or (??) is satisfied, we say that the species balance condition for *S*_2_ is satisfied.

Even though species balance conditions for *S*_2_and *S*_3_ are satisfied, the limit of the normalized species numbers for *S*_2_or *S*_3_ may become degenerate. Consider addition of species *S*_2_and *S*_3_ as a single virtual species, and compare the collective rates of production and consumption of this species. Recall that *S*_23_ denotes addition of species *S*_2_and *S*_3_. Since production of one species is canceled by consumption of the other species, maximal production rate of *S*_23_ may be different from that of *S*_2_or *S*_3_. Suppose that the maximal collective rates of production or consumption of *S*_23_ are slower than the maximal production or consumption rates of *S*_2_and *S*_3_. Also, suppose that the maximal collective rates of production and consumption of the complex have different orders of magnitude. Then, a limit of the normalized species number of *S*_23_can be zero or infinity, even though the species balance conditions for *S*_2_ and *S*_3_ are satisfied. Therefore, we need an additional condition to obtain balance between collective production and consumption rates for *S*_23_. To obtain a balance equation for *S*_23_, we unnormalize (11a) and (11b) by multiplying *N*^*α*^_2_ and *N*^*α*^_3_, respectively. Adding the unnormalized equations for species *S*_2_and *S*_3_ and dividing it by $N^{\max(\alpha_{2},\alpha_{3})}$, we get

(15) $$\;\begin{array}{l} N^{-\max(\alpha_{2},\alpha_{3})}\left(N^{\alpha_{2}}Z_{2}^{N,\gamma }(t)+N^{\alpha_{3}}Z_{3}^{N,\gamma }(t)\right)\\ \;=N^{-\max(\alpha_{2},\alpha_{3})}\;\left(\;N^{\alpha_{2}}Z_{2}^{N,\gamma }(0)+N^{\alpha_{3}}Z_{3}^{N,\gamma }(0)\;\right)\\ \;+N^{-\max(\alpha_{2},\alpha_{3})}R_{4}^{t}(N^{\gamma +\rho_{4}}\kappa_{4}Z_{1}^{N,\gamma })\\ \;+N^{-\max(\alpha_{2},\alpha_{3})}R_{8}^{t}(N^{\gamma +\rho_{8}}\kappa_{8}Z_{7}^{N,\gamma })\\ \;-N^{-\max(\alpha_{2},\alpha_{3})}R_{9}^{t}(N^{\gamma +\rho_{9}}\kappa_{9}Z_{2}^{N,\gamma }Z_{6}^{N,\gamma }). \end{array}$$

Comparing the maximal scaling exponents of production and consumption of *S*_23_ in (15), a balance equation for *S*_23_is given as

(16) $$\max(\rho_{4},\rho_{8})=\rho_{9}.$$

In case (16) is not satisfied, the order of magnitude of the species number for *S*_23_ should be larger than those of collective production and consumption rates so that a difference between production and consumption is not noticeable. This gives

(17) $$\max(\alpha_{2},\alpha_{3})\geq \gamma +\max(\rho_{4},\rho_{8},\rho_{9}).$$

Solving (??) for *γ*, we get

(18) $$\gamma \leq \max(\alpha_{2},\alpha_{3})-\max(\rho_{4},\rho_{8},\rho_{9})\equiv u_{23}.$$

Similarly to the time-scale constraint in the species balance condition, (18) implies that if maximal collective production and consumption rates for *S*_23_are not balanced, our choice of values for scaling exponents are valid up to times of order *N*^*u*^_23_.

We call (16) and (18) the *collective species balance condition* for *S*_23_, that is, either (16) or (18) must hold. The species balance conditions for all species and the collective species balance conditions for all positive linear combinations of species should be satisfied to obtain a nondegenerate limit of $Z_{i}^{N,\gamma }$ (Condition 3.2 in [9]). Condition 3.2 can be reduced by Lemma 3.4-3.8 and Remark 3.9 in [9]. A key idea is to find a minimum subset of linear combinations of species so that production of one species is canceled by consumption of the other species when we combine the species. In that case, maximal collective production rate of the linear combination of the species may be different from that of each species. Therefore, species balance conditions may not imply the collective species balance condition for the linear combination of the species. For example, a collective species balance condition for addition of *S*_2_ and *S*_3_ should be satisfied, since reactions producing *S*_2_or *S*_3_ may not increase the species number of *S*_23_. In Table 4, we choose linear combinations of species whose collective species balance conditions may not be satisfied by the species balance conditions. For other linear combinations of species, their collective species balance conditions are derived from the ones in Table 4. Satisfying all balance conditions in Table 4 guarantees satisfying balance conditions for all positive linear combination of species, and these conditions help to identify scaling exponents which give a nondegenerate limit of the normalized species numbers in the heat shock response model of *E. coli*. In most cases satisfying balance conditions gives nondegenerate limiting models in the times of interest, but we can still find counter examples as given in the last paragraph in the section for methods.

**Table 4:** Balance equations and time-scale constraints for each species and for each collective species chosen

	**Balance equations**	**Time-scale constraints**
*S*_1_	*ρ*_13_=*ρ*_14_	$\gamma \leq \alpha_{1}-\max(\rho_{13},\rho_{14})$
*S*_2_	$\max(\rho_{3},\rho_{4},\rho_{5},\rho_{6},\rho_{7},\rho_{8})=\max(\rho_{2},\rho_{9})$	$\gamma \leq \alpha_{2}-\max(\rho_{2},\rho_{3},\rho_{4},\rho_{5},\rho_{6},\rho_{7},\rho_{8},\rho_{9})$
*S*_3_	$\rho_{2}=\max(\rho_{3},\rho_{5},\rho_{6},\rho_{7})$	$\gamma \leq \alpha_{3}-\max(\rho_{2},\rho_{3},\rho_{5},\rho_{6},\rho_{7})$
*S*_4_	*ρ*_6_=*ρ*_18_	$\gamma \leq \alpha_{4}-\max(\rho_{6},\rho_{18})$
*S*_5_	*ρ*_5_=*ρ*_16_	$\gamma \leq \alpha_{5}-\max(\rho_{5},\rho_{16})$
*S*_6_	$\max(\rho_{7},\rho_{8},\rho_{12},\rho_{15})=\max(\rho_{9},\rho_{10},\rho_{17})$	$\gamma \leq \alpha_{6}-\max(\rho_{7},\rho_{8},\rho_{9},\rho_{10},\rho_{12},\rho_{15},\rho_{17})$
*S*_7_	$\rho_{9}=\max(\rho_{8},\rho_{15})$	$\gamma \leq \alpha_{7}-\max(\rho_{8},\rho_{9},\rho_{15})$
*S*_8_	$\max(\rho_{1},\rho_{12})=\max(\rho_{10},\rho_{11})$	$\gamma \leq \alpha_{8}-\max(\rho_{1},\rho_{10},\rho_{11},\rho_{12})$
*S*_9_	*ρ*_10_=*ρ*_12_	$\gamma \leq \alpha_{9}-\max(\rho_{10},\rho_{12})$
*S*_2_ + *S*_3_ + *S*_7_	*ρ*_4_=*ρ*_15_	$\gamma \leq \max(\alpha_{2},\alpha_{3},\alpha_{7})-\max(\rho_{4},\rho_{15})$
*S*_2_ + *S*_3_	$\max(\rho_{4},\rho_{8})=\rho_{9}$	$\gamma \leq \max(\alpha_{2},\alpha_{3})-\max(\rho_{4},\rho_{8},\rho_{9})$
*S*_2_ + *S*_7_	$\max(\rho_{3},\rho_{4},\rho_{5},\rho_{6},\rho_{7})=\max(\rho_{2},\rho_{15})$	$\gamma \leq \max(\alpha_{2},\alpha_{7})-\max(\rho_{2},\rho_{3},\rho_{4},\rho_{5},\rho_{6},\rho_{7},\rho_{15})$
*S*_6_ + *S*_7_ + *S*_9_	*ρ*_7_=*ρ*_17_	$\gamma \leq \max(\alpha_{6},\alpha_{7},\alpha_{9})-\max(\rho_{7},\rho_{17})$
*S*_6_ + *S*_7_	$\max(\rho_{7},\rho_{12})=\max(\rho_{10},\rho_{17})$	$\gamma \leq \max(\alpha_{6},\alpha_{7})-\max(\rho_{7},\rho_{10},\rho_{12},\rho_{17})$
*S*_6_ + *S*_9_	$\max(\rho_{7},\rho_{8},\rho_{15})=\max(\rho_{9},\rho_{17})$	$\gamma \leq \max(\alpha_{6},\alpha_{9})-\max(\rho_{7},\rho_{8},\rho_{9},\rho_{15},\rho_{17})$
*S*_8_ + *S*_9_	*ρ*_1_=*ρ*_11_	$\gamma \leq \max(\alpha_{8},\alpha_{9})-\max(\rho_{1},\rho_{11})$

In each case, either the balance equation or the time-scale constraint must hold.

Based on species and collective species balance equations in Table 4, we choose appropriate values for *α*_*i*_’s and *β*_*k*_’s so that most of the balance equations are satisfied. If some of the balance equations are not satisfied, corresponding time-scale constraints give a range of *γ* where the chosen *α*_*i*_’s and *β*_*k*_’s are valid. The time-scale constraint, *γ*≤*γ*_0_, implies that the set of scaling exponents *α*_*i*_’s and *β*_*k*_’s chosen is appropriate only up to time whose order of magnitude is equal to *N*^*γ*^_0_. For the times larger than *O*(*N*^*γ*^_0_), we need to choose a different set of values for the scaling exponents, *α*_*i*_’s. Assuming that reaction rate constants do not change in time and that the species numbers vary in time, we in general use one set of *β*_*k*_’s for all time scales and may use several sets of *α*_*i*_’s. A large change of the species numbers in time requires different *α*_*i*_’s in different time scales. For the heat shock model we identify three different time scales as we will see in the section of limiting models in three time scales, and *α*_1_, *α*_2_, *α*_3_, *α*_8_, and *α*_9_ may depend on the time scale. *α*_4_, *α*_5_, *α*_6_, and *α*_7_ are the same for all time scales.

Before we determine scaling exponents for *S*_1_, *S*_2_, and *S*_3_, we run one realization of stochastic simulation to find ranges of the species numbers in time. Using initial values for species *S*_1_, *S*_2_, and *S*_3_, *X*_1_(0)=10 and *X*_2_(0)=*X*_3_(0)=1 as given in Table 1 and using *N*_0_=100, we set $X_{1}(t)\approx O(100)=O(N_{0}^{\alpha_{1}})$, $X_{2}(t)\approx O(1)=O(N_{0}^{\alpha_{2}})$, and $X_{3}(t)\approx O(1)=O(N_{0}^{\alpha_{3}})$ with *α*_1_=1 and *α*_2_=*α*_3_=0 in the early stage of time period. Plugging in *α*_*i*_’s and *β*_*k*_’s in the balance equations for *S*_2_, *S*_3_, and *S*_23_, equality holds in (12a) and (12b) but not in (16). Therefore, (18) gives

$$\begin{array}{lll} \gamma & \leq \max(\alpha_{2},\alpha_{3})-\max(\rho_{4},\rho_{8},\rho_{9})\; & \;\\ & =\max(0,0)-\max(0,-2,-2)=0.\; & \; \end{array}$$

Then, the first set of scaling exponents with *α*_1_=1 and *α*_2_=*α*_3_=0 is valid only when *γ*≤0. Next, based on the fact that *X*_2_(*t*)≈*O*(10) and *X*_3_(*t*)≈*O*(10) in the medium stage of time period, we choose *α*_2_=*α*_3_=0 for *γ*>0. At this stage of time period, we set $X_{1}(t)=O(10)\approx O(N_{0}^{\alpha_{1}})$ with *α*_1_=0. Then, (12a) and (12b) are satisfied but not (16). The condition (18) gives *γ*≤1, and the second set of scaling exponents with *α*_1_=*α*_2_=*α*_3_=0 is valid when *γ*≤1. Finally, we set *α*_1_=0 and *α*_2_=*α*_3_=1 for *γ*>1 based on the fact that the numbers of molecules of *S*_2_and *S*_3_ grow in time and are of order 100. Then, (12a), (12b), and (16) are all satisfied, and the third set of scaling exponents with *α*_1_=0 and *α*_2_=*α*_3_=1 can be used for *γ*>1.

The three sets of values for the scaling exponents chosen are given in the Additional file 1: Table S4. With chosen values for the scaling exponents, we check whether each balance equation is satisfied and give a time-scale constraint in the Additional file 1: Table S6 in case the balance equation is not satisfied. Different choices of *α*_*i*_’s and *β*_*k*_’s from the ones in the Additional file 1: Table S4 give different limiting models. As long as the chosen values for *α*_*i*_’s and *β*_*k*_’s satisfy balance conditions, the limiting model will describe nontrivial behavior of the species numbers which are nonzero and finite in the specific time of interest.

### Limiting models in three time scales

In the heat shock response model of *E. coli*, we identify a time scale of interest using the chosen set of scaling exponents and derive a limiting model which approximates dynamics of the full chemical reaction network. Each limiting model involves a subset of species and reactions, and gives features of the full network during the time interval of interest.

To identify a time scale involving a limiting model with interesting dynamics (nondegenerate), we first need to determine a natural time scale of each species. Recall that a natural time scale of species *S*_*i*_ is the time period of order *N*^*γ*^_*i*_ when $Z_{i}^{N,\gamma_{i}}(t)$ is of order 1. The natural time scale exponent *γ*_*i*_for species *S*_*i*_ is rigorously determined by

(19) $$\underset{k\in \Gamma_{i}^{+}\cup \Gamma_{i}^{-}}{\max}(\gamma_{i}+\rho_{k})=\alpha_{i},$$

where *Γi* + denotes the collection of reactions where the species number of *S*_*i*_ increases every time the reaction occurs. Similarly, *Γi*− is the subset of reactions where the species number of *S*_*i*_decreases every time the reaction occurs. In (19), the left-side term is the maximal order of magnitude of rates of reactions involving *S*_*i*_and the right-side term is the order of magnitude of the species number for *S*_*i*_. If times are earlier than those of order *N*^*γ*^_*i*_(*γ*<*γ*_*i*_), fluctuations of species number of *S*_*i*_ due to the reactions involving *S*_*i*_are not noticeable compared to magnitude of the species number of *S*_*i*_. Then, the species number of *S*_*i*_ is approximated as its initial value. In the times of order *N*^*γ*^_*i*_(*γ*=*γ*_*i*_), changes of species number of *S*_*i*_ due to the reactions and the species number of *S*_*i*_ are similar in magnitude and behavior of the species number of *S*_*i*_is described by its nondegenerate limit. If times are later than those of order *N*^*γ*^_*i*_(*γ*>*γ*_*i*_), the species number of *S*_*i*_ fluctuates very rapidly due to the reactions involving *S*_*i*_ compared to the magnitude of the species number of *S*_*i*_. Then, the averaged behavior of the species number of *S*_*i*_is approximated by some function of other species numbers. Note that *γ*_*i*_ depends on *α*_*i*_’s and *β*_*k*_’s, and the time scale of the *i*th species may change if we use several sets of *α*_*i*_’s.

All values of *α*_*i*_’s and *ρ*_*k*_’s for three scalings which are used to derive limiting models are given in the Additional file 1: Table S4. The equations for normalized species numbers and the equation for $Z_{23}^{N,\gamma }$ which are used later in this section are given in the Additional file 1: Section 1 and Section 2, respectively. When we derive limiting models in three time scales, boundedness of the normalized species numbers is required. For first two time scales, we define stopping times so that the normalized species numbers are bounded up to those times. For the last time scale, we proved stochastic boundedness of some normalized species numbers in a finite time interval. For more details, see Additional file 1: Section 5.

Consider a model with the first set of scaling exponents including *α*_1_=1 and *α*_2_=*α*_3_=0. Note that the first set of scaling exponents is valid when *γ*≤0 based on the time scale constraints given in Table 4. Substituting *α*_2_=0 and *ρ*_*k*_’s for the first scaling to the equation for $Z_{2}^{N,\gamma }$ given in (11a), we have

(20) $$\;\begin{array}{l} Z_{2}^{N,\gamma }(t)=Z_{2}^{N,\gamma }(0)+R_{3}^{t}\left(N^{\gamma }\kappa_{3}Z_{3}^{N,\gamma }\right)+R_{4}^{t}\left(N^{\gamma }\kappa_{4}Z_{1}^{N,\gamma }\right)\\ \;+R_{5}^{t}\left(N^{\gamma -1}\kappa_{5}Z_{3}^{N,\gamma }\right)+R_{6}^{t}\left(N^{\gamma -1}\kappa_{6}Z_{3}^{N,\gamma }\right)\\ \;+R_{7}^{t}\left(N^{\gamma -1}\kappa_{7}Z_{3}^{N,\gamma }\right)+R_{8}^{t}\left(N^{\gamma -2}\kappa_{8}Z_{7}^{N,\gamma }\right)\\ \;-R_{2}^{t}\left(N^{\gamma }\kappa_{2}Z_{2}^{N,\gamma }\right)-R_{9}^{t}\left(N^{\gamma -2}\kappa_{9}Z_{2}^{N,\gamma }Z_{6}^{N,\gamma }\right). \end{array}$$

When *γ*=*γ*_2_, the maximal scaling exponent in the propensities of all reaction terms in (20) should be equal to the scaling exponent for the species number of *S*_2_. Therefore, *γ*_2_satisfies

(21) $$\max(\gamma_{2},\gamma_{2}-1,\gamma_{2}-2)=0=\alpha_{2},$$

and we get *γ*_2_=0. Similarly, we get *γ*_3_=*γ*_8_=0.

Next, we plug *α*_1_=1 and *ρ*_*k*_’s for the first scaling in the equation for $Z_{1}^{N,\gamma }$ and get

(22) $$\;\begin{array}{l} Z_{1}^{N,\gamma }(t)=Z_{1}^{N,\gamma }(0)+N^{-1}R_{13}^{t}\left(N^{\gamma -2}\kappa_{13}\right)\\ \;-N^{-1}R_{14}^{t}\left(N^{\gamma -1}\kappa_{14}Z_{1}^{N,\gamma }\right). \end{array}$$

By comparing the maximal scaling exponent in the propensities of all reaction terms in (22) and the scaling exponent for the species number of *S*_1_, *γ*_1_ satisfies

(23) $$\max(\gamma_{1}-2,\gamma_{1}-1)=1=\alpha_{1},$$

and we get *γ*_1_=2. Similarly, we get *γ*_*i*_>0 for *i*=4,5,6,7,9. Among all natural time scale exponents of species, we choose the smallest one, *γ*=0, and set *t*∼*O*(*N*^0^)=*O*(1) as the first time scale we are interested in. Since *γ*_1_>0, $Z_{1}^{N,0}(t)\to Z_{1}^{0}(0)$ as *N*→*∞*. Similarly, $Z_{i}^{N,0}(t)\to Z_{i}^{0}(0)=N_{0}^{-\alpha_{i}}X_{i}(0)$ for *i*=4,5,6,7,9 as *N*→*∞*. To sum up, in this time scale with *γ*=0, the species numbers of *S*_*i*_’s for *i*=1,4,5,6,7,9 change more slowly than other species numbers, and the species numbers with slow time scales are approximated as constant.

To derive the limiting equation for *S*_2_, we set *γ*=0 in (20). Since the 2nd, 3rd, and 4th reaction terms have propensities with *N*^0^=1 and the species number of *S*_2_ is of order 1, these reaction terms converge to nonzero limits in the limiting equation. On the other hand, the propensities of the 5th, 6th, 7th, 8th and 9th reaction terms are of order *N*^−1^ or *N*^−2^ which are smaller than the species number for *S*_2_of order 1. Therefore, these reaction terms converge to zero as *N*→*∞*at least in the finite time interval. In the 2nd and 3rd reaction terms in (20), $Z_{2}^{N,0}(s)\to Z_{2}^{0}(s)$ and $Z_{3}^{N,0}(s)\to Z_{3}^{0}(s)$ as *N*→*∞* since *γ*_2_=*γ*_3_=0. Then, using $Z_{1}^{N,0}(s)\to Z_{1}^{0}(0)$ as *N*→*∞*, the limit of $Z_{2}^{N,0}$ satisfies

$$Z_{2}^{0}(t)=Z_{2}^{0}(0)+R_{3}^{t}\left(\kappa_{3}Z_{3}^{0}\right)+R_{4}^{t}\left(\kappa_{4}Z_{1}^{0}(0)\right)-R_{2}^{t}\left(\kappa_{2}Z_{2}^{0}\right).$$

Similarly, we get a limiting model with $Z_{2}^{0}$, $Z_{3}^{0}$, and $Z_{8}^{0}$ for *γ*=0 as given in (3).

Next, consider a model with the second set of scaling exponents including *α*_1_=*α*_2_=*α*_3_=0. Note that the second set of scaling exponents is valid when *γ*≤1 based on the time scale constraints given in Table 4. To determine the natural time scale of *S*_6_, substitute *α*_6_=0 and *ρ*_*k*_’s for the second scaling in the equation for $Z_{6}^{N,\gamma }$, and we have

(24) $$\;\begin{array}{l} Z_{6}^{N,\gamma }(t)=Z_{6}^{N,\gamma }(0)+R_{7}^{t}\left(N^{\gamma -1}\kappa_{7}Z_{3}^{N,\gamma }\right)\\ \;+R_{8}^{t}\left(N^{\gamma -2}\kappa_{8}Z_{7}^{N,\gamma }\right)+R_{12}^{t}\left(N^{\gamma -1}\kappa_{12}Z_{9}^{N,\gamma }\right)\\ \;+R_{15}^{t}\left(N^{\gamma -1}\kappa_{15}Z_{4}^{N,\gamma }Z_{7}^{N,\gamma }\right)\\ \;-R_{9}^{t}\left(N^{\gamma -2}\kappa_{9}Z_{2}^{N,\gamma }Z_{6}^{N,\gamma }\right)\\ \;-R_{10}^{t}\left(N^{\gamma -1}\kappa_{10}Z_{6}^{N,\gamma }Z_{8}^{N,\gamma }\right)\\ \;-R_{17}^{t}\left(N^{\gamma -2}\kappa_{17}Z_{6}^{N,\gamma }\right). \end{array}$$

Comparing the exponents inside and outside of the reaction terms in (24), *γ*_6_ satisfies

(25) $$\max(\gamma_{6}-1,\gamma_{6}-2)=0=\alpha_{6},$$

and we get *γ*_6_=1. Similarly, we get *γ*_7_=*γ*_8_=1, *γ*_*i*_<1 for *i*=2,3, and *γ*_*i*_>1 for *i*=1,4,5,9. We already get the temporal behavior of species numbers of *S*_2_, *S*_3_, and *S*_8_ through the limiting model when *γ*=0. Thus, we set *t*∼*O*(*N*^1^) as the second time scale we are interested in, and derive a limiting model for *S*_6_, *S*_7_, and *S*_8_ when *γ*=1. Note that species *S*_8_ is involved in the limiting models for both *γ*=0 and *γ*=1, since we use different sets of scaling exponents in these models. For *i*=1,4,5,9$Z_{i}^{N,1}(t)\to Z_{i}^{1}(0)$ as *N*→*∞*, since *γ*_*i*_>1. Thus, in the 12th and 15th reaction terms in (24), $Z_{9}^{N,1}(s)\to Z_{9}^{1}(0)$ and $Z_{4}^{N,1}(s)\to Z_{4}^{1}(0)$ as *N*→*∞*. Since the propensities of the 8th, 9th, and 17th reaction terms in (24) are of order *N*^*γ*−2^=*N*^−1^ for *γ*=1 and the species number of *S*_6_ is of order 1, these reaction terms go to zero as *N*→*∞*. In the 10th and 15th reaction terms in (24), $Z_{6}^{N,1}(s)$, $Z_{7}^{N,1}(s)$, and $Z_{8}^{N,1}(s)$ are asymptotically *O*(1) and converge to $Z_{6}^{1}(s)$, $Z_{7}^{1}(s)$, and $Z_{8}^{1}(s)$ as *N*→*∞* since *γ*_6_=*γ*_7_=*γ*_8_=1.

Now, consider the asymptotic behavior of the 7th reaction term in (24) when *γ*=1. Since *γ*_3_<1, $Z_{3}^{N,1}(t)$ fluctuates very much, and there exists no functional limit as *N*→*∞*. However, $\overset{t}{\underset{0}{\int }}Z_{3}^{N,1}(s)\;\mathrm{ds}$ still converges, which gives the averaged behavior of the normalized species number of *S*_3_. To get the limit of $\overset{t}{\underset{0}{\int }}Z_{3}^{N,1}(s)\;\mathrm{ds}$, we plug the second set of scaling exponents in the equation for $Z_{3}^{N,\gamma }$ and obtain

(26) $$\;\begin{array}{l} Z_{3}^{N,1}(t)=Z_{3}^{N,1}(0)+R_{2}^{t}\left(N\kappa_{2}Z_{2}^{N,1}\right)-R_{3}^{t}\left(N\kappa_{3}Z_{3}^{N,1}\right)\\ \;-R_{5}^{t}\left(\kappa_{5}Z_{3}^{N,1}\right)-R_{6}^{t}\left(\kappa_{6}Z_{3}^{N,1}\right)-R_{7}^{t}\left(\kappa_{7}Z_{3}^{N,1}\right). \end{array}$$

The law of large numbers of Poisson processes gives an asymptotic limit of the scaled reaction terms as

(27) $$\underset{N\to \infty }{\lim}\underset{u\leq u_{0}}{\sup}\left|\frac{Y_{k}(N^{\alpha_{i}}u)}{N^{\alpha_{i}}}-u\right|=0,\;u_{0}>0$$

where the *Y*_*k*_’s are unit Poisson processes and *α*_*i*_>0. For example, the 2nd reaction term in (26) divided by *N* is approximated as

$$\;\frac{R_{2}^{t}(N\kappa_{2}Z_{2}^{N,1})}{N}=\frac{Y_{2}(\int_{0}^{t}N\kappa_{2}Z_{2}^{N,1}(s)\;\mathrm{ds})}{N}\;\approx \;\int_{0}^{t}\kappa_{2}Z_{2}^{N,1}\left(s\right)\;\mathrm{ds.}$$

Dividing (26) by *N* and using the law of large numbers for Poisson processes, we get

(28) $$\int_{0}^{t}\left(\kappa_{2}Z_{2}^{N,1}(s)-\kappa_{3}Z_{3}^{N,1}(s)\right)\;\mathrm{ds}\to 0,$$

as *N*→*∞*.

We introduce an auxiliary variable to make the limiting model closed and define

$$Z_{23}^{N,\gamma }(t)\equiv Z_{2}^{N,\gamma }(t)+Z_{3}^{N,\gamma }(t).$$

Plugging *α*_2_=*α*_3_=0 and *ρ*_*k*_’s in the second scaling in the equation for $Z_{23}^{N,\gamma }$, we get

(29) $$\;\begin{array}{l} Z_{23}^{N,\gamma }(t)=Z_{23}^{N,\gamma }(0)\;+\;R_{4}^{t}\left(\;N^{\gamma -1}\kappa_{4}Z_{1}^{N,\gamma }\;\right)\;+\;R_{8}^{t}\left(\;N^{\gamma -2}\kappa_{8}Z_{7}^{N,\gamma }\;\right)\\ \;-R_{9}^{t}\left(N^{\gamma -2}\kappa_{9}Z_{2}^{N,\gamma }Z_{6}^{N,\gamma }\right). \end{array}$$

Since $Z_{23}^{N,\gamma_{23}}(t)∼O(1)$ where *γ*_23_ denotes a natural time scale exponent of *S*_23_, we compare the scaling exponents of *N* in the reaction terms in (29) and the scaling exponent of *N* outside the reaction terms. Then *γ*_23_satisfies

$$\max(\gamma_{23}-1,\gamma_{23}-2)=0=\max(\alpha_{2},\alpha_{3}),$$

and we get *γ*_23_=1. Since these reaction terms have *N*^*γ*−2^=*N*^−1^ in their propensities when *γ*=1, which is smaller than the species number for *S*_23_ of order 1, these reaction terms converge to zero as *N*→*∞*. Using $Z_{1}^{N,1}(s)\to Z_{1}^{1}(0)$, the limit of $Z_{23}^{N,1}$ satisfies

$$Z_{23}^{1}(t)=Z_{23}^{1}(0)+R_{4}^{t}\left(\kappa_{4}Z_{1}^{1}(0)\right).$$

Adding and subtracting terms in (28) and dividing the equation by −(*κ*_2_ + *κ*_3_), we get

$$\int_{0}^{t}\left(Z_{3}^{N,1}\left(s\right)-\frac{\kappa_{2}}{\kappa_{2}+\kappa_{3}}Z_{23}^{N,1}\left(s\right)\right)\;\mathrm{ds}\to 0,$$

as *N*→*∞*, and this is used to obtain the limit of the 7th reaction term in (24). Letting *N*→*∞*, the limiting equation for $Z_{6}^{N,1}$ is given as

(30) $$\begin{array}{l} Z_{6}^{1}(t)=Z_{6}^{1}(0)+R_{7}^{t}\left(\frac{\kappa_{2}\kappa_{7}}{\kappa_{2}+\kappa_{3}}Z_{23}^{1}\right)+R_{12}^{t}\left(\kappa_{12}Z_{9}^{1}\left(0\right)\right)\\ \;+R_{15}^{t}\left(\kappa_{15}Z_{4}^{1}\left(0\right)Z_{7}^{1}\right)-R_{10}^{t}\left(\kappa_{10}Z_{6}^{1}Z_{8}^{1}\right). \end{array}$$

In (30), note that $R_{12}^{t}(\kappa_{12}Z_{9}^{1}(0))=0$ since *X*_9_(0)=0 as given in Table 1. Limiting equations for $Z_{7}^{N,1}$ and $Z_{8}^{N,1}$ can be derived similarly, and a limiting model with $Z_{23}^{1}$, $Z_{6}^{1}$, $Z_{7}^{1}$, and $Z_{8}^{1}$ for *γ*=1 is given in (4).

Last, consider a model with the third scaling exponents with *α*_1_=0 and *α*_2_=*α*_3_=1. To derive a limiting equation for $Z_{23}^{N,2}$, we plug *ρ*_*k*_’s and *α*_2_=*α*_3_=1 for the third scaling in the equation for $Z_{23}^{N,\gamma }$ and get

(31) $$\;\begin{array}{l} Z_{23}^{N,2}(t)=Z_{23}^{N,2}(0)+N^{-1}\left[R_{4}^{t}\left(N\kappa_{4}Z_{1}^{N,2}\right)+R_{8}^{t}\left(\kappa_{8}Z_{7}^{N,2}\right)\right)\\ \;-\left(R_{9}^{t}\left(N\kappa_{9}Z_{2}^{N,2}Z_{6}^{N,2}\right)\right]. \end{array}$$

In (31), the 8th reaction term is asymptotically zero, since the term is of order *N*^−1^. Using the law of large numbers for Poisson processes in (27), the 4th and the 9th terms in (31) are asymptotically equal to

(32) $$\int_{0}^{t}\left(\kappa_{4}Z_{1}^{N,2}\left(s\right)-\kappa_{9}Z_{2}^{N,2}\left(s\right)Z_{6}^{N,2}\left(s\right)\right)\;\mathrm{ds.}$$

Since *γ*_1_=2, $Z_{1}^{N,2}(s)\to Z_{1}^{2}(s)$ as *N*→*∞*. On the other hand, since *γ*_2_,*γ*_6_<2, both $Z_{2}^{N,2}(s)$ and $Z_{6}^{N,2}(s)$ in (32) fluctuate rapidly and we must identify the averaged limit. $Z_{3}^{N,2}$ is also averaged, since *γ*_3_<2. We actually show convergence of the fast-fluctuating species numbers of *S*_2_ and *S*_3_ to some limits in the Additional file 1: Section 5.1. For any *ε*>0 and for any *t* such that $\varepsilon <t\leq \tau_{\infty }^{2}$,

(33) $$Z_{2}^{N,2}(t)\to {\bar{Z}}_{2}^{2}(t),$$

(34) $$Z_{3}^{N,2}(t)\to {\bar{Z}}_{3}^{2}(t),$$

uniformly as *N*→*∞*.

On the other hand, since *γ*_6_<2, $\overset{t}{\underset{0}{\int }}Z_{6}^{N,2}(s)\;\mathrm{ds}$ converges to a limit which gives averaged behavior of the normalized species number of *S*_6_. Using the equation for $Z_{6}^{N,\gamma }$, we get

(35) $$\;\begin{array}{l} Z_{6}^{N,2}(t)=Z_{6}^{N,2}(0)+R_{7}^{t}(N^{2}\kappa_{7}Z_{3}^{N,2})+R_{8}^{t}(\kappa_{8}Z_{7}^{N,2})\\ \;+R_{12}^{t}(N^{2}\kappa_{12}Z_{9}^{N,2})+R_{15}^{t}(N\kappa_{15}Z_{4}^{N,2}Z_{7}^{N,2})\\ \;-R_{9}^{t}(N\kappa_{9}Z_{2}^{N,2}Z_{6}^{N,2})-R_{10}^{t}(N^{2}\kappa_{10}Z_{6}^{N,2}Z_{8}^{N,2})\\ \;-R_{17}^{t}(\kappa_{17}Z_{6}^{N,2}). \end{array}$$

Dividing (35) by *N*^2^, using the law of large numbers for Poisson processes in (27), and using the stochastic boundedness of the propensities of the 8th, 9th, 15th, and 17th reaction terms in the finite time interval shown in the Additional file 1: Section 5.1, we get

(36) $$\;\int_{0}^{t}\;\left(\kappa_{7}Z_{3}^{N,2}\left(s\right)\;+\;\kappa_{12}Z_{9}^{N,2}\left(s\right)\;-\;\kappa_{10}Z_{6}^{N,2}\left(s\right)Z_{8}^{N,2}\left(s\right)\right)\mathrm{ds}\to 0,$$

as *N*→*∞*. Therefore, a difference between the 10th and 12th reaction terms is approximated in terms of

(37) $$\int_{0}^{t}\kappa_{7}Z_{3}^{N,2}\left(s\right)\;\mathrm{ds},$$

which converges to $\int_{0}^{t}\kappa_{7}{\bar{Z}}_{3}^{2}(s)\;\mathrm{ds}$ from (34). Therefore, we get

(38) $$\;\int_{0}^{t}\;\left(\kappa_{10}Z_{6}^{N,2}\left(s\right)Z_{8}^{N,2}\left(s\right)\;-\;\kappa_{12}Z_{9}^{N,2}\left(s\right)\right)\mathrm{ds}\;\to \overset{t}{\underset{0}{\int }}\kappa_{7}{\bar{Z}}_{3}^{2}\left(s\right)\;\mathrm{ds},$$

as *N*→*∞*.

Now, from the equations for $Z_{8}^{N,\gamma }$ and $Z_{9}^{N,\gamma }$, we get

(39a) $$\;\begin{array}{l} Z_{8}^{N,2}(t)=Z_{8}^{N,2}(0)+N^{-2}\left[R_{1}^{t}\left(N^{2}\kappa_{1}\right)+R_{12}^{t}\left(N^{2}\kappa_{12}Z_{9}^{N,2}\right)\right)\\ \;-\left(R_{10}^{t}\left(N^{2}\kappa_{10}Z_{6}^{N,2}Z_{8}^{N,2}\right)-R_{11}^{t}\left(N^{2}\kappa_{11}Z_{8}^{N,2}\right)\right], \end{array}$$

(39b) $$\begin{array}{l} Z_{9}^{N,2}(t)=Z_{9}^{N,2}(0)+N^{-2}\left[R_{10}^{t}\left(N^{2}\kappa_{10}Z_{6}^{N,2}Z_{8}^{N,2}\right)\right)\\ \;-\left(R_{12}^{t}\left(N^{2}\kappa_{12}Z_{9}^{N,2}\right)\right]. \end{array}$$

Using the law of large numbers of Poisson processes in (27), the reaction terms in (39a) and (39b) are asymptotically equal to

(40a) $$\;\begin{array}{l} N^{-2}\left[R_{1}^{t}\left(N^{2}\kappa_{1}\right)\;+\;R_{12}^{t}\;\left(\;N^{2}\kappa_{12}Z_{9}^{N,2}\;\right)\;-\;R_{10}^{t}\;\left(N^{2}\kappa_{10}Z_{6}^{N,2}Z_{8}^{N,2}\;\right)\right)\\ \;-\left(R_{11}^{t}\left(N^{2}\kappa_{11}Z_{8}^{N,2}\right)\right]\\ \;\approx \int_{0}^{t}\left(\kappa_{1}+\kappa_{12}Z_{9}^{N,2}(s)-\kappa_{10}Z_{6}^{N,2}(s)Z_{8}^{N,2}(s)\right)\\ \;\left(-\kappa_{11}Z_{8}^{N,2}(s)\right)\;\mathrm{ds}, \end{array}$$

(40b) $$\;\begin{array}{l} N^{-2}\left[R_{10}^{t}(N^{2}\kappa_{10}Z_{6}^{N,2}Z_{8}^{N,2})-R_{12}^{t}(N^{2}\kappa_{12}Z_{9}^{N,2})\right]\\ \;\approx \int_{0}^{t}\left(\kappa_{10}Z_{6}^{N,2}(s)Z_{8}^{N,2}(s)-\kappa_{12}Z_{9}^{N,2}(s)\right)\;\mathrm{ds.} \end{array}$$

Using (40a), (40b), and (38), the limiting equations of (39a) and (39b) are given as

(41) $$\begin{array}{lll} Z_{8}^{2}(t) & =Z_{8}^{2}(0)+\int_{0}^{t}\left(\kappa_{1}-\kappa_{7}{\bar{Z}}_{3}^{2}(s)-\kappa_{11}Z_{8}^{2}(s)\right)\;\mathrm{ds},\; & \;\\ Z_{9}^{2}(t) & =Z_{9}^{2}(0)+\int_{0}^{t}\kappa_{7}{\bar{Z}}_{3}^{2}(s)\;\mathrm{ds.}\; \end{array}$$

In (41), note that $Z_{9}^{2}(0)=0$ since *X*_9_(0)=0 as given in Table 1.

Since $Z_{8}^{N,2}(0)>0$ and balance conditions are satisfied, $Z_{8}^{N,2}(t)\neq 0$ in the finite time interval. Since *γ*_8_=2,

(42) $$\frac{1}{\kappa_{10}Z_{8}^{N,2}(t)}\to \frac{1}{\kappa_{10}Z_{8}^{2}(t)}.$$

Using (38) and (42), $\int_{0}^{t}Z_{6}^{N,2}(s)\;\mathrm{ds}$ is averaged as

(43) $$\int_{0}^{t}Z_{6}^{N,2}(s)\;\mathrm{ds}\to \int_{0}^{t}\frac{\kappa_{7}{\bar{Z}}_{3}^{2}(s)+\kappa_{12}Z_{9}^{2}(s)}{\kappa_{10}Z_{8}^{2}(s)}\;\mathrm{ds.}$$

From (33) and (43), we get

(44) $$\;\begin{array}{l} \int_{0}^{t}\kappa_{9}Z_{2}^{N,2}(s)Z_{6}^{N,2}(s)\;\mathrm{ds}\to \int_{0}^{t}\kappa_{9}{\bar{Z}}_{2}^{2}(s)\\ \;\times \left(\frac{\kappa_{7}{\bar{Z}}_{3}^{2}(s)+\kappa_{12}Z_{9}^{2}(s)}{\kappa_{10}Z_{8}^{2}(s)}\right)\;\mathrm{ds.} \end{array}$$

For more details used in (43) and (44), see Lemma 1.5 and Theorem 2.1 in [15]. Finally, we get the limiting equation of $Z_{23}^{N,2}$ as

$$\begin{array}{l} Z_{23}^{2}(t)=Z_{23}^{2}(0)+\int_{0}^{t}\left[\kappa_{4}Z_{1}^{2}\left(s\right)-\kappa_{9}{\bar{Z}}_{2}^{2}\left(s\right)\right)\\ \;\times \left(\left(\frac{\kappa_{7}{\bar{Z}}_{3}^{2}\left(s\right)+\kappa_{12}Z_{9}^{2}\left(s\right)}{\kappa_{10}Z_{8}^{2}\left(s\right)}\right)\right]\;\mathrm{ds.} \end{array}$$

#### Theorem 1

For *γ*=0, $\left\{Z_{2}^{N,0},Z_{3}^{N,0},Z_{8}^{N,0}\right\}$ converges to the solution of (3) for $t\in [0,\tau_{\infty }^{0})$. For *γ*=1, $\left\{Z_{23}^{N,1},Z_{6}^{N,1},Z_{7}^{N,1},Z_{8}^{N,1}\right\}$ converges to the solution of (4) for $t\in [0,\tau_{\infty }^{1})$. In (3), $Z_{8}^{0}$ is a discrete process, while $Z_{8}^{1}$ is a deterministic process in (4). For *γ*=2, $\left\{Z_{1}^{N,2},Z_{23}^{N,2},Z_{4}^{N,2},Z_{5}^{N,2},Z_{8}^{N,2},Z_{9}^{N,2}\right\}$ converges to the solution of (5) for $t\in [0,\tau_{\infty }^{2})$.

### Conditional equilibrium distributions

In the previous section, we derived limiting models in three different time scales. Except for the subset of species in the limiting model, the remaining species are approximated as constants in the first time scale, since their natural time scale exponents (*γ*_*i*_) are larger than *γ*=0, i.e., species with *γ*_*i*_>*γ*=0 did not start to fluctuate at these times yet. In the second and third time scales, there are subsets of species whose natural time scale exponents are smaller than *γ*=1 and 2, respectively. Normalized species numbers with *γ*_*i*_<*γ*fluctuate very rapidly at these times and their averaged behavior is approximated in terms of other variables which converge to a nondegenerate limit. For those species, the normalized species numbers do not converge to a limit in a functional sense, but still we can find a limit in a probabilistic sense (i.e. convergence in distribution) and their distribution. Conditioned on the normalized species numbers which converge to a nondegenerate limit in the time scale of interest, we can find the *conditional equilibrium* (or the *local averaging*) distributions of species numbers whose natural time scale exponents are smaller than the time scale exponents of interests. Conditioning on the normalized species numbers which converge to a nondegenerate limit is similar to fixing slowly-moving variables and describing behavior of the fast-fluctuating variables in terms of slowly-moving variables treating them as constants. In the next remark, we give a conditional equilibrium distribution of the subset of species with natural time scale exponents smaller than *γ*=1 and *γ*=2.

#### Remark 2

For *γ*=1, for each *t*>0, $\left(Z_{2}^{N,1}(t),Z_{3}^{N,1}(t)\right)$ converges in distribution to $\left({\hat{Z}}_{2}^{1}(t),{\hat{Z}}_{3}^{1}(t)\right)$ such that $\left({\hat{Z}}_{2}^{1}(t),{\hat{Z}}_{3}^{1}(t)\right)$ conditioned on $Z_{23}^{1}(t)$ has a binomial distribution with parameter

$$\frac{\kappa_{3}}{\kappa_{2}+\kappa_{3}},\;\frac{\kappa_{2}}{\kappa_{2}+\kappa_{3}},$$

respectively, that is,

$$\;\begin{array}{l} P\left\{{\hat{Z}}_{2}^{1}(t)=z_{2},{\hat{Z}}_{3}^{1}(t)=m-z_{2}|Z_{23}^{1}(t)=m\right\}\\ \;=C(m,z_{2}){\left(\frac{\kappa_{3}}{\kappa_{2}+\kappa_{3}}\right)}^{z_{2}}{\left(\frac{\kappa_{2}}{\kappa_{2}+\kappa_{3}}\right)}^{m-z_{2}}. \end{array}$$

For *γ*=2, for each *t*>0, $\left(Z_{6}^{N,2}(t),Z_{7}^{N,2}(t)\right)$ converges in distribution to $\left({\hat{Z}}_{6}^{2}(t),{\hat{Z}}_{7}^{2}(t)\right)$ where ${\hat{Z}}_{6}^{2}(t)$ and ${\hat{Z}}_{7}^{2}(t)$ are independent Poisson distributed random variables with parameters

$$\frac{\frac{\kappa_{2}\kappa_{7}}{\kappa_{2}+\kappa_{3}}Z_{23}^{2}(t)+\kappa_{12}Z_{9}^{2}(t)}{\kappa_{10}Z_{8}^{2}(t)},$$

and

$$\frac{\frac{\kappa_{3}\kappa_{9}}{\kappa_{2}+\kappa_{3}}Z_{23}^{2}(t)}{\kappa_{15}Z_{4}^{2}(t)}\cdot \frac{\frac{\kappa_{2}\kappa_{7}}{\kappa_{2}+\kappa_{3}}Z_{23}^{2}(t)+\kappa_{12}Z_{9}^{2}(t)}{\kappa_{10}Z_{8}^{2}(t)}.$$

The detailed method to compute conditional equilibrium distributions is given in Section 6 in *[*[9]*]*.

Mean value of the random variable with a binomial distribution, *B*(*n*,*p*), is equal to *np*. Therefore, for *γ*=1, we treat $Z_{23}^{1}(t)$ as constant and get a limit of the averaged values for $Z_{2}^{N,1}(t)$ and $Z_{3}^{N,1}(t)$ as

$$\begin{array}{lll} {\bar{Z}}_{2}^{1}(t) & =Z_{23}^{1}(t)\times \frac{\kappa_{3}}{\kappa_{2}+\kappa_{3}},\; & \;\\ {\bar{Z}}_{3}^{1}(t) & =Z_{23}^{1}(t)\times \frac{\kappa_{2}}{\kappa_{2}+\kappa_{3}}.\; & \; \end{array}$$

Mean value of the random variable with a Poisson distribution, *Pois*(*λ*), is equal to *λ*, and we obtain a limit of the averaged values for $Z_{6}^{N,2}(t)$ and $Z_{7}^{N,2}(t)$ as the parameters given in Remark 2.

### Simulation results

Recall that the normalized species numbers after a time change are defined as

$$Z_{i}^{N,\gamma }(t)=N^{-\alpha_{i}}X_{i}^{N}(tN^{\gamma }).$$

**Figure 2:**
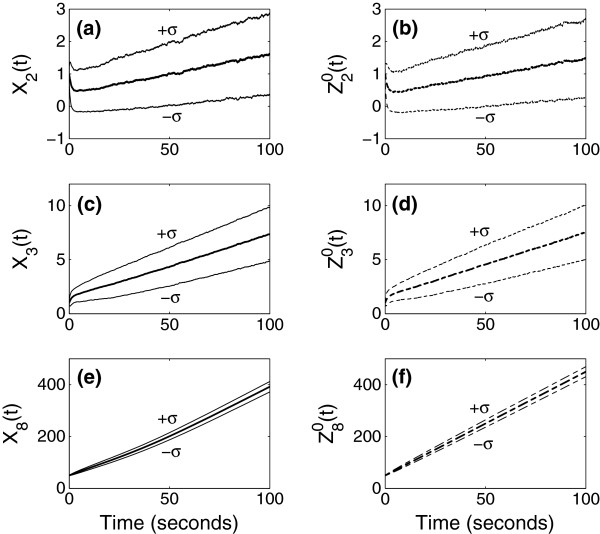
**Simulation results when *γ *= 0.** Simulation of the full model (left) and that of approximation using the limiting model (right) when the time is of order $N_{0}^{0}$ (=1).

**Figure 3:**
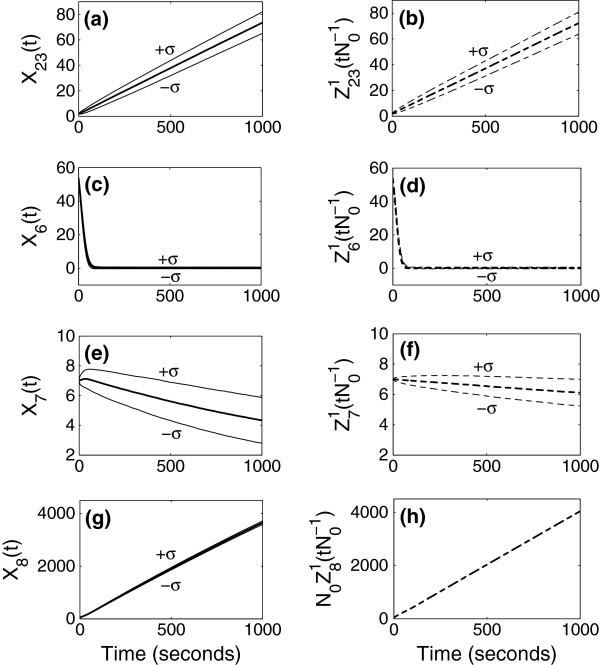
**Simulation results when *γ * = 1.** Simulation of the full model (left) and that of approximation using the limiting model (right) when the time is of order *N*_0_(=100).

**Figure 4:**
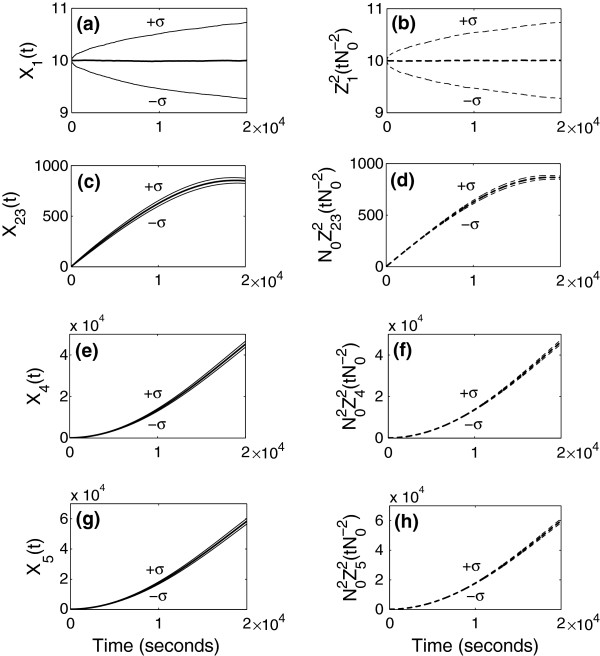
**Simulation results when *γ *= 2.** Simulation of the full model (left) and that of approximation using the limiting model (right) when the time is of order $N_{0}^{2}$ (=10000).

**Figure 5:**
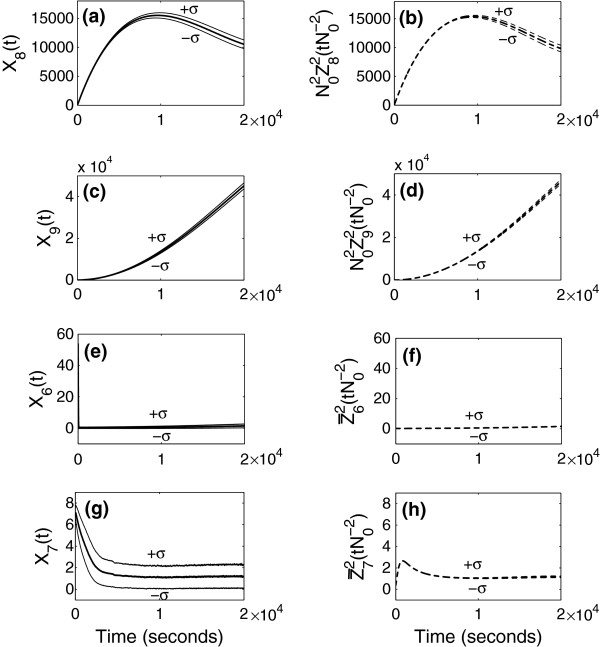
**Simulation results when *γ *= 2 (continued).** Simulation of the full model (left) and that of approximation using the limiting model (right) when the time is of order $N_{0}^{2}$. Figures (e), (f), (g), and (h) are simulation results for species 6 and 7. The graphs (f) and (h) give approximation of the averaged species numbers of *S*_6_and *S*_7_.

Using the limiting models in the three time scales given in (3)-(5), we approximate the species numbers in the full model by unnormalizing the species numbers and applying time change backward as

$$\begin{array}{l} X_{i}(t)=X_{i}^{N_{0}}(t)\approx \underset{N\to \infty }{\lim}{\left(\frac{N_{0}}{N}\right)}^{\alpha_{i}}X_{i}^{N}\left(t{\left(\frac{N}{N_{0}}\right)}^{\gamma }\right)\\ \;=N_{0}^{\alpha_{i}}Z_{i}^{\gamma }(tN_{0}^{-\gamma }), \end{array}$$

using a real value *N*_0_=100 for the parameter. In Figures 2, 3, 4 and Figure 5(a)-(d), the panels located in the left column give mean and standard deviation from the mean of stochastic simulation for *X*_*i*_(*t*) and the panels located in the right column give mean and standard deviation from the mean of simulation for $N_{0}^{\alpha_{i}}Z_{i}^{\gamma }(tN_{0}^{-\gamma })$ using the limiting models. The mean and standard deviation of species numbers are computed from 3000 realizations of the sample path of the stochastic simulation.

In Figure 2, we compare the simulation for the full model and for the approximation using the limiting model in the first scaling. The first scaling (*γ*=0) is for the times of order $N_{0}^{0}=1\sec$, and we look at the evolution of mean and standard deviation of the species numbers up to 100 sec. The full model and the limiting model for *γ*=0 are stochastic, and the limiting model approximates the evolution of statistics of the species numbers quite precisely. As shown in Figure 2(f) $Z_{8}^{0}(t)$ overestimates *X*_8_(*t*), since the limiting model does not include reactions consuming *S*_8_. Therefore, consumption of *S*_8_may not be captured well in the approximation.

In Figure 3, we compare the simulation for the full model and for the approximation using the limiting model in the second scaling. Since the second scaling (*γ*=1) is for the times of order $N_{0}^{1}=100\sec$, we observe the evolution of the species numbers up to 1000 sec. In this time scale, the evolution of *S*_8_shown in Figure 3(h) is approximated by a deterministic variable. The evolution of the species number of *S*_8_ in the full model given in Figure 3(g) is stochastic, but its standard deviation is very small. As in the previous time scale, $N_{0}Z_{8}^{1}(tN_{0}^{-1})$ slightly overestimates *X*_8_(*t*), since the limiting model does not include any consumptions of *S*_8_. The remaining three species, *S*_23_, *S*_6_, and *S*_7_are approximated by stochastic variables. The increasing species number of *S*_23_ in time and the rapid decrease in species number of *S*_6_are well captured by the limiting model. The species numbers of *S*_7_are described by stochastic variables both in the full model and in the limiting model. The behavior of *S*_7_in two models is not exactly the same, and discrepancy of the mean species numbers of *S*_7_ comes from the approximation of *X*_4_(*t*) in terms of its initial value. In the limiting model, *S*_7_is approximated as a stochastic process decreasing by 1 with the propensity proportional to $Z_{4}^{1}(0)$. However, *X*_4_(*t*) increases during the times in [0,1000] sec in the full model, and this difference gives slower decreasing rate of the mean number of *S*_7_ in the limiting model than that in the full model.

In Figure 4 and Figure 5(a)-(d), we compare the simulation for the full model and for the approximation obtained from the limiting model in the third scaling. Since the third scaling (*γ*=2) is for the times of order $N_{0}^{2}=10,000\sec$, we look at the simulation up to 20,000 sec. In this time scale, the limiting model is stochastic. The species number of *S*_1_ in the limiting model is approximated by a stochastic discrete variable increasing and decreasing by 1, and the remaining species numbers in the limiting model satisfy stochastic equations driven by the stochastic discrete variable $Z_{1}^{2}$. As we have seen in the proof of Theorem 1 in the Additional file 1: Section 5.1, the processes for *S*_1_ in the full model and in the limiting model are exactly the same. Therefore, we use a same series of random numbers, when we simulate the full and limiting models. In Figure 4(b), the process for *S*_1_ is random, but its standard deviation is very small. Therefore, in one realization of simulation of the limiting model, behavior of *S*_1_appears as constant. Since all the remaining variables in the limiting model are governed by the variable for *S*_1_ and they satisfy the stochastic differential equations, evolution of one sample path of the species numbers for *S*_23_, *S*_4_, *S*_5_, *S*_8_, and *S*_9_ in the limiting model looks like a solution of the system of ordinary differential equations.

In Figure 5, (e)-(h) are the species numbers for *S*_6_and *S*_7_in the full model and their averaged values in the limiting model. Note that the species numbers for *S*_6_ and *S*_7_ do not appear in the limiting model, since their values are approximated in terms of other species numbers. Therefore, the difference between mean species numbers for *S*_6_and *S*_7_in the full model and those in the approximation does not affect the error directly. For *γ*=2, $Z_{6}^{N,2}$ and $Z_{7}^{N,2}$ are asymptotically averaged out by the variables in the limiting model as given in Remark 2. Since the averaged value for *S*_6_ plays an important role in the evolution of $Z_{23}^{2}$ in the limiting model and since the averaged value for *S*_7_ gives the conditional mean value for *S*_7_ in the limiting model, we compare the species numbers of *S*_6_and *S*_7_ in the full model and the approximated averaged values in the limiting model. In Figure 5(f) and (h), we plot the mean and standard deviation from the mean for

$$\;\begin{array}{l} {\bar{Z}}_{6}^{2}\;\left(tN_{0}^{-2}\right)=\frac{\frac{\kappa_{2}\kappa_{7}}{\kappa_{2}\;+\;\kappa_{3}}Z_{23}^{2}\left(tN_{0}^{-2}\right)\;+\;\kappa_{12}Z_{9}^{2}\;\left(\;tN_{0}^{-2}\right)}{\kappa_{10}Z_{8}^{2}\left(tN_{0}^{-2}\right)},\\ {\bar{Z}}_{7}^{2}\;\left(tN_{0}^{-2}\right)=\frac{\kappa_{9}\;\left(\;\frac{\kappa_{3}}{\kappa_{2}\;+\;\kappa_{3}}Z_{23}^{2}\left(tN_{0}^{-2}\;\right)\;\right)\;\left(\;\frac{\frac{\kappa_{2}\kappa_{7}}{\kappa_{2}\;+\;\kappa_{3}}Z_{23}^{2}\left(tN_{0}^{-2}\;\right)\;+\;\kappa_{12}Z_{9}^{2}\left(tN_{0}^{-2}\right)}{\kappa_{10}Z_{8}^{2}\left(tN_{0}^{-2}\;\right)}\;\right)}{\kappa_{15}Z_{4}^{2}\;\left(\;tN_{0}^{-2}\;\right)}, \end{array}$$

in time. They are stochastic variables determined by the ones in the limiting model with very small fluctuations. Since ${\bar{Z}}_{6}^{2}(tN_{0}^{-2})$ and ${\bar{Z}}_{7}^{2}(tN_{0}^{-2})$ describe averaged behavior of *S*_6_and *S*_7_, *X*_6_(*t*) and *X*_7_(*t*) in Figure 5(e) and (g) have more fluctuations than the averaged species numbers in Figure 5(f) and (h).

In Figure 5(e)-(h) there is a discrepancy between the species numbers and their averaged values in the very early time, and the discrepancy comes from a disagreement in initial values of the species numbers in the full model and those of the averaged values in the limiting model. The integrated species numbers for *S*_6_ and *S*_7_ up to times of order 10,000 are supposed to be approximated by the integrated averaged values over the time interval, and the initial difference is due to a boundary layer phenomenon.

### Error estimates

In the previous sections, we scaled species numbers and derived their limit to approximate temporal behavior of the species numbers in the full network. Among three limiting models given in (3)-(5), the first two are systems with discrete variables (except for $Z_{8}^{1}$) which change by integer values. On the other hand, the last one is a hybrid system with both discrete and continuous variables. A discrete variable $Z_{1}^{2}$ increases or decreases by one and stochasticity of all other variables comes from how much $Z_{1}^{2}$ fluctuates. Since $Z_{1}^{2}$ rarely changes at the times of our interest, the rest of the variables in (5) behaves such as a solution of systems of ordinary differential equations. Our choice of the scaling parameter value, *N*_0_=100, is not very large and it is possible that the limiting model does not contain enough fluctuations as much as the full network actually has due to our assumption that *N*_0_is replaced by a large parameter *N*.

In this section, we estimate an error between the normalized species numbers and their limit given in (5) at the times of 10,000 sec. Define

$$\;\begin{array}{l} U^{N}\left(t\right)=N^{1/2}\left(Z_{23}^{N,2}\left(t\right)-Z_{23}^{2}\left(t\right),Z_{4}^{N,2}\left(t\right)-Z_{4}^{2}\left(t\right),Z_{5}^{N,2}\left(t\right)\right)\\ \;-{\left(Z_{5}^{2}\left(t\right),Z_{8}^{N,2}\left(t\right)-Z_{8}^{2}\left(t\right),Z_{9}^{N,2}\left(t\right)-Z_{9}^{2}\left(t\right)\right)}^{T}, \end{array}$$

and denote *U*(*t*)=(*U*_23_(*t*),*U*_4_(*t*),*U*_5_(*t*),*U*_8_(*t*),*U*_9_(*t*))^*T*^ as a limit of *U*^*N*^(*t*) as *N* goes to infinity. Note that we do not consider an error between $Z_{1}^{N,2}(t)$ and $Z_{1}^{2}(t)$, since they are exactly the same processes. In the next remark, we show that *U*^*N*^(*t*) converges to *U*(*t*) in the probabilistic sense and thus the error between $Z_{i}^{N,2}(t)$ and $Z_{i}^{2}(t)$ is approximately of order $N_{0}^{-1/2}=0.1$. Since *U*(*t*) gives an explicit form of the error, we have better approximation of *X*_*i*_(*t*) for *γ*=2 as

$$X_{i}\left(t\right)\approx N_{0}^{\alpha_{i}}\left(Z_{i}^{2}\left(tN_{0}^{-2}\right)+N_{0}^{-1/2}U_{i}\left(tN_{0}^{-2}\right)\right),$$

for *S*_23_, *S*_4_, *S*_5_, *S*_8_, and *S*_9_.

#### Remark 3

For *γ*=2, for each *t*>0, *U*^*N*^(*t*) converges in distribution to *U*(*t*) which is a solution of

$$\;\begin{array}{l} U(t)=U(0)\;+\;\overset{t}{\underset{0}{\int }}{\left(1,0,0,0,0\right)}^{T}\;\sqrt{\kappa_{4}Z_{1}^{2}(s)\;+\;\kappa_{9}{\bar{Z}}_{2}^{2}(s){\bar{Z}}_{6}^{2}(s)}\;\mathrm{dW}(s)\\ \;+\int_{0}^{t}\left[\begin{array}{l} C_{23}(s)U_{23}(s)+C_{8}(s)U_{8}(s)+C_{9}(s)U_{9}(s)\\ \;\frac{\kappa_{2}\kappa_{6}}{\kappa_{2}+\kappa_{3}}U_{23}(s)-\kappa_{18}U_{4}(s)\\ \;\frac{\kappa_{2}\kappa_{5}}{\kappa_{2}+\kappa_{3}}U_{23}(s)-\kappa_{16}U_{5}(s)\\ \;-\frac{\kappa_{2}\kappa_{7}}{\kappa_{2}+\kappa_{3}}U_{23}(s)-\kappa_{11}U_{8}(s)\\ \;\frac{\kappa_{2}\kappa_{7}}{\kappa_{2}+\kappa_{3}}U_{23}(s) \end{array}\right]\;\mathrm{ds}, \end{array}$$

where *W*(*t*) is a standard Brownian motion and

$$\begin{array}{lll} C_{23}\left(s\right) & =-\frac{\kappa_{9}}{\kappa_{2}+\kappa_{3}}\left(\kappa_{3}{\bar{Z}}_{6}^{2}\left(s\right)+\frac{\kappa_{2}\kappa_{7}}{\kappa_{10}}\cdot \frac{{\bar{Z}}_{2}^{2}\left(s\right)}{Z_{8}^{2}\left(s\right)}\right),\; & \;\\ C_{8}\left(s\right) & =\kappa_{9}\frac{{\bar{Z}}_{2}^{2}\left(s\right){\bar{Z}}_{6}^{2}\left(s\right)}{Z_{8}^{2}\left(s\right)},\; & \;\\ C_{9}\left(s\right) & =-\frac{\kappa_{9}\kappa_{12}}{\kappa_{10}}\cdot \frac{{\bar{Z}}_{2}^{2}\left(s\right)}{Z_{8}^{2}\left(s\right)}.\; & \; \end{array}$$

The detailed method to compute an error using the central limit theorem is derived in [14].

Estimating order of magnitude of an error is an analogue of that in van Kampen’s system size expansion [16]. A difference is that in the system size expansion, the system state representing the species numbers is scaled by the system size *Ω* and noise between the scaled process and its deterministic value is approximated as a random variable of order *Ω*^−1/2^. In our approach *N* is not a system size but a parameter for scaling, and species numbers are scaled by powers of *N*. Though the limiting model for *γ*=2 is not deterministic, it is still possible to estimate an error analytically due to the fact that $Z_{1}^{2}(t)$ which produces stochasticity in the limiting model is an exact process equal to $Z_{1}^{N,2}(t)$. Another difference between our approach and van Kampen’s system size expansion is that a subset of species numbers is averaged in terms of other species numbers which appear in the limiting model for *γ*=2 due to the various scales involved.

Our estimates of the error is also different from diffusion approximations. In the diffusion approximations, the reaction terms centered by their propensities in the stochastic equations for discrete variables of species numbers are approximated in terms of time-changed Brownian motion. On the other hand, the noise term in the error estimates is determined by both the centered reaction terms in the equations for discrete variables and a difference between the discrete variables for the normalized species number and their continuous limit.

To find the asymptotic order of magnitude of $Z_{i}^{N,2}(t)-Z_{i}^{2}(t)$, we show convergence of $r_{N}\left(Z_{i}^{N,2}(t)-Z_{i}^{2}(t)\right)$ to a nonzero finite limit for some *r*_*N*_. Among the species *S*_23_, *S*_4_, *S*_5_, *S*_8_, and *S*_9_, the species number of *S*_23_is scaled with the smallest exponent, and thus noise in the limit of $r_{N}\left(Z_{i}^{N,2}(t)-Z_{i}^{2}(t)\right)$ is determined dominantly by the component $r_{N}\left(Z_{23}^{N,2}(t)-Z_{23}^{2}(t)\right)$. Since $Z_{23}^{N,2}(t)$ is the species number scaled by *N*, we expect that *r*_*N*_=*N*^1/2^and the error between the scaled species numbers and their limit is of order $N_{0}^{-1/2}$. For a detailed approach to derive *r*_*N*_and *U*(*t*), see more about the central limit theorem in [14]. The fact that all components but the first one in the diffusion term in the equation for *U*(*t*) are zero supports the idea that noise is dominantly determined by the error between $Z_{23}^{N,2}(t)$ and $Z_{23}^{2}(t)$. A sketch of the proof of Remark 3 is given in the Additional file 1: Section 6.

## Conclusions

We considered a stochastic model for a well-stirred biochemical network with small numbers of molecules for some species. As the biochemical network consists of more species and reactions, network topology becomes more complex and it is harder to analyze. Therefore, how to reduce the biochemical network while preserving its important biochemical features is a very important issue.

In this paper, we applied the multiscale approximation method introduced by Ball et al. [8] and extended by Kang and Kurtz [9] to a heat shock response model of *E. coli* developed by Srivastava et al. [11]. Using the fact that the species numbers and the reaction rate constants in the model vary over several orders of magnitude, we scaled them using a scaling parameter with different exponents both of which contribute to determining the time scales of species. We derived balance conditions for each species and for a subset of linear combinations of species explicitly in this model, and chose appropriate values for the scaling exponents satisfying the balance conditions. Assuming that initial values of the species numbers are positive, satisfying the balance conditions is required to get a nondegenerate limiting model. We assumed that the reaction rate constants do not change in time, while we may use several sets of scaling exponents for the species numbers due to rapid changes in some species numbers in time. In this analysis, we chose three sets of scaling exponents, and they are used to derive limiting models in different time scales.

In each time scale we derived a limiting model, and used it to approximate the species numbers in the full network. In the limiting model, species numbers whose scaling exponents are larger than those of all rates of reactions involving the species are treated as constants, since changes of the species numbers due to the reactions are not noticeable at these times. When the scaling exponent of the species number is smaller than the scaling exponents of the rates of some productions and consumptions of the species and in case the scaling exponents for both kinds of reactions are equal, the scaled species number is averaged out and is approximated in terms of other variables. Therefore, the limiting model includes a subset of species and reactions and network topology in it becomes simpler. We derived the conditional equilibrium distributions of the fast-fluctuating species numbers and studied errors between the scaled species numbers and their limits in the third time scale.

Using the limiting models, we approximated the temporal evolution of species numbers in three time scales. By comparing stochastic simulation of the full model and approximations using the limiting models, we see that the main features of evolution of species numbers are well captured by the limiting models.

## Competing interests

The author(s) declare that they have no competing interests.

## Authors’ contributions

Based on the model of heat shock response of *E. coli* developed in [11], the author applied the multiscale approximation method introduced in [9] to the model. The author derived limiting models, showed convergence of the scaled species numbers to their limits, and estimated errors analytically. The author simulated the full network model and approximate processes using the limiting models and compared the results.

## Supplementary Material

Additional file 1 **Supplementary material for “A multiscale approximation in a heat shock response model of E. coli.”** This is a supplementary material of the paper including calculations and tables. Click here for file

## References

[B1] Kærn M Elston T Blakem W Collins J Stochasticity in gene expression: from theories to phenotypes Nat Rev Genet 2005 6 6 451 464 15883588 10.1038/nrg1615

[B2] Gillespie D A general method for numerically simulating the stochastic time evolution of coupled chemical reactions J Comput Phys 1976 22 4 403 434

[B3] Gillespie D Exact stochastic simulation of coupled chemical reactions J Phys Chem 1977 81 25 2340 2361

[B4] Rao C Arkin A Stochastic chemical kinetics and the quasi-steady-state assumption: application to the Gillespie algorithm J Chem Phys 2003 118 11 4999 5010

[B5] Haseltine E Rawlings J Approximate simulation of coupled fast and slow reactions for stochastic chemical kinetics J Chemi Phys 2002 117 15 6959 6969

[B6] Cao Y Gillespie D Petzold L Multiscale stochastic simulation algorithm with stochastic partial equilibrium assumption for chemically reacting systems J Comput Phys 2005 206 2 395 411

[B7] Pahle J Biochemical simulations: stochastic, approximate stochastic and hybrid approaches Briefings Bioinf 2009 10 1 53 64 10.1093/bib/bbn050 PMC2638628 19151097

[B8] Ball K Kurtz T Popovic L Rempala G Asymptotic analysis of multiscale approximations to reaction networks Ann Appl Probability 2006 16 4 1925 1961

[B9] Kang HW Kurtz T Separation of time-scales and model reduction for stochastic reaction networks 2012 arXiv preprint arXiv:1011.1672, to appear in Annals of Applied Probability

[B10] Crudu A Debussche A Radulescu O Hybrid stochastic simplifications for multiscale gene networks BMC Syst Biol 2009 3 89 19735554 10.1186/1752-0509-3-89 PMC2761401

[B11] Srivastava R Peterson M Bentley W Stochastic kinetic analysis of the Escherichia coli stress circuit using σ32-targeted antisense Biotechnol Bioeng 2001 75 1 120 129 11536134 10.1002/bit.1171

[B12] Takahashi K Kaizu K Hu B Tomita M A multi-algorithm, multi-timescale method for cell simulation Bioinformatics 2004 20 4 538 546 14990450 10.1093/bioinformatics/btg442

[B13] Weinan E Vanden-Eijnden E Nested stochastic simulation algorithm for chemical kinetic systems with disparate rates J Chem Phys 2005 123 19 194107 16321076 10.1063/1.2109987

[B14] Kang HW Popovic L Kurtz T Central limit theorems and diffusion approximations for multiscale Markov chain models 2012 arXiv preprint arXiv:1208.3783, submitted

[B15] Kurtz T Averaging for martingale problems and stochastic approximation Appl Stochastic Anal 1992 186 209

[B16] Van Kampen NG Stochastic processes in physics and chemistry (North-Holland Personal Library) 2007 Elsevier

