# Supplementary material for: A multiscale approximation in a heat shock response model of E. coli
Source: BMC Syst Biol. 2012 Nov 21;6:143. doi: 10.1186/1752-0509-6-143 (PMC3608964; doi:10.1186/1752-0509-6-143)
Supplement: Additional file 1 — Supplementary material for “A multiscale approximation in a heat shock response model of E. coli.” This is a supplementary material of the paper including calculations and tables. [file 1752-0509-6-143-S1.pdf]

Supplementary material for "A multiscale approximation in a heat shock response model of E. coli"

Hye-Won Kang*

[1] Mathematical Biosciences Institute, Ohio State University, Columbus, OH, USA

Email: kang.235@mbi.osu.edu;

*Corresponding author

1 The scaled stochastic equations

The stochastic equation governing the species numbers is

$$X(t) = X(0) + \sum_{k=1}^{r_0} R_k^t(\lambda_k(X))(\nu_k' - \nu_k),$$

where

$$R_k^t(\lambda_k(X)) = Y_k\left(\int_0^t \lambda_k(X(s))\,ds\right),$$

and the Y_k's are independent unit Poisson processes. Let Λ_N be an $s_0 \times s_0$-dimensional diagonal matrix with entries $N^{-\alpha_i}$. The process for the scaled species numbers after a time change is described by

$$Z^{N,\gamma}(t) = Z^{N,\gamma}(0) + \Lambda_N \sum_{k=1}^{r_0} R_k^t\left(N^{\gamma+\rho_k}\hat{\lambda}_k(Z^{N,\gamma})\right)(\nu_k' - \nu_k).$$

The process $Z^{N,\gamma}$ is an s_0-dimensional vector with each component written as

$$\begin{aligned}
Z_1^{N,\gamma}(t) &= Z_1^{N,\gamma}(0) + N^{-\alpha_1}\Big[R_{13}^t(N^{\gamma+\rho_{13}}\kappa_{13}) - R_{14}^t(N^{\gamma+\rho_{14}}\kappa_{14}Z_1^{N,\gamma})\Big],\\
Z_2^{N,\gamma}(t) &= Z_2^{N,\gamma}(0) + N^{-\alpha_2}\Big[R_3^t(N^{\gamma+\rho_3}\kappa_3 Z_3^{N,\gamma}) + R_4^t(N^{\gamma+\rho_4}\kappa_4 Z_1^{N,\gamma})\\
&\quad + R_5^t(N^{\gamma+\rho_5}\kappa_5 Z_3^{N,\gamma}) + R_6^t(N^{\gamma+\rho_6}\kappa_6 Z_3^{N,\gamma}) + R_7^t(N^{\gamma+\rho_7}\kappa_7 Z_3^{N,\gamma})\\
&\quad + R_8^t(N^{\gamma+\rho_8}\kappa_8 Z_7^{N,\gamma}) - R_2^t(N^{\gamma+\rho_2}\kappa_2 Z_2^{N,\gamma})\\
&\quad - R_9^t(N^{\gamma+\rho_9}\kappa_9 Z_2^{N,\gamma} Z_6^{N,\gamma})\Big],\\
Z_3^{N,\gamma}(t) &= Z_3^{N,\gamma}(0) + N^{-\alpha_3}\Big[R_2^t(N^{\gamma+\rho_2}\kappa_2 Z_2^{N,\gamma}) - R_3^t(N^{\gamma+\rho_3}\kappa_3 Z_3^{N,\gamma})\\
&\quad - R_5^t(N^{\gamma+\rho_5}\kappa_5 Z_3^{N,\gamma}) - R_6^t(N^{\gamma+\rho_6}\kappa_6 Z_3^{N,\gamma}) - R_7^t(N^{\gamma+\rho_7}\kappa_7 Z_3^{N,\gamma})\Big],
\end{aligned}$$

$$
\begin{aligned}
Z_4^{N,\gamma}(t) &= Z_4^{N,\gamma}(0) + N^{-\alpha_4}\Big[R_6^t(N^{\gamma+\rho_6}\kappa_6 Z_3^{N,\gamma}) - R_{18}^t(N^{\gamma+\rho_{18}}\kappa_{18} Z_4^{N,\gamma})\Big], \\
Z_5^{N,\gamma}(t) &= Z_5^{N,\gamma}(0) + N^{-\alpha_5}\Big[R_5^t(N^{\gamma+\rho_5}\kappa_5 Z_3^{N,\gamma}) - R_{16}^t(N^{\gamma+\rho_{16}}\kappa_{16} Z_5^{N,\gamma})\Big], \\
Z_6^{N,\gamma}(t) &= Z_6^{N,\gamma}(0) + N^{-\alpha_6}\Big[R_7^t(N^{\gamma+\rho_7}\kappa_7 Z_3^{N,\gamma}) + R_8^t(N^{\gamma+\rho_8}\kappa_8 Z_7^{N,\gamma}) \\
&\quad + R_{12}^t(N^{\gamma+\rho_{12}}\kappa_{12} Z_9^{N,\gamma}) + R_{15}^t(N^{\gamma+\rho_{15}}\kappa_{15} Z_4^{N,\gamma} Z_7^{N,\gamma}) \\
&\quad - R_9^t(N^{\gamma+\rho_9}\kappa_9 Z_2^{N,\gamma} Z_6^{N,\gamma}) - R_{10}^t(N^{\gamma+\rho_{10}}\kappa_{10} Z_6^{N,\gamma} Z_8^{N,\gamma}) \\
&\quad - R_{17}^t(N^{\gamma+\rho_{17}}\kappa_{17} Z_6^{N,\gamma})\Big], \\
Z_7^{N,\gamma}(t) &= Z_7^{N,\gamma}(0) + N^{-\alpha_7}\Big[R_9^t(N^{\gamma+\rho_9}\kappa_9 Z_2^{N,\gamma} Z_6^{N,\gamma}) - R_8^t(N^{\gamma+\rho_8}\kappa_8 Z_7^{N,\gamma}) \\
&\quad - R_{15}^t(N^{\gamma+\rho_{15}}\kappa_{15} Z_4^{N,\gamma} Z_7^{N,\gamma})\Big], \\
Z_8^{N,\gamma}(t) &= Z_8^{N,\gamma}(0) + N^{-\alpha_8}\Big[R_1^t(N^{\gamma+\rho_1}\kappa_1) + R_{12}^t(N^{\gamma+\rho_{12}}\kappa_{12} Z_9^{N,\gamma}) \\
&\quad - R_{10}^t(N^{\gamma+\rho_{10}}\kappa_{10} Z_6^{N,\gamma} Z_8^{N,\gamma}) - R_{11}^t(N^{\gamma+\rho_{11}}\kappa_{11} Z_8^{N,\gamma})\Big], \\
Z_9^{N,\gamma}(t) &= Z_9^{N,\gamma}(0) + N^{-\alpha_9}\Big[R_{10}^t(N^{\gamma+\rho_{10}}\kappa_{10} Z_6^{N,\gamma} Z_8^{N,\gamma}) - R_{12}^t(N^{\gamma+\rho_{12}}\kappa_{12} Z_9^{N,\gamma})\Big].
\end{aligned}
$$

In each reaction term, R_k^t, the propensity includes $N^{\gamma+\rho_k}$ produced from scaling the species numbers in the propensity and from change of the time variable. ρ_k's are given in the following table in terms of α_i's and β_k's.

Table 1: Scaling exponents in propensities

ρ_k		ρ_k		ρ_k	
ρ_1	β_1	ρ_7	$\alpha_3 + \beta_7$	ρ_{13}	β_{13}
ρ_2	$\alpha_2 + \beta_2$	ρ_8	$\alpha_7 + \beta_8$	ρ_{14}	$\alpha_1 + \beta_{14}$
ρ_3	$\alpha_3 + \beta_3$	ρ_9	$\alpha_2 + \alpha_6 + \beta_9$	ρ_{15}	$\alpha_4 + \alpha_7 + \beta_{15}$
ρ_4	$\alpha_1 + \beta_4$	ρ_{10}	$\alpha_6 + \alpha_8 + \beta_{10}$	ρ_{16}	$\alpha_5 + \beta_{16}$
ρ_5	$\alpha_3 + \beta_5$	ρ_{11}	$\alpha_8 + \beta_{11}$	ρ_{17}	$\alpha_6 + \beta_{17}$
ρ_6	$\alpha_3 + \beta_6$	ρ_{12}	$\alpha_9 + \beta_{12}$	ρ_{18}	$\alpha_4 + \beta_{18}$

2 Identities

In this section, the governing equations for the linear combinations of the species used in this paper are given. Denote addition of species S_2 and S_3 as S_{23}, addition of species S_6 and S_7 as S_{67}, addition of S_2, S_3, and S_7 as S_{237}, and addition of S_6, S_7, and S_9 as S_{679}. Define variables for the normalized numbers of the

linear combinations of the species, S_{23}, S_{67}, S_{237}, and S_{679} as

$$\begin{aligned}
Z_{23}^{N,\gamma}(t) &\equiv \frac{N^{\alpha_2}Z_2^{N,\gamma}(t)+N^{\alpha_3}Z_3^{N,\gamma}(t)}{N^{\max(\alpha_2,\alpha_3)}},\\
Z_{67}^{N,\gamma}(t) &\equiv \frac{N^{\alpha_6}Z_6^{N,\gamma}(t)+N^{\alpha_7}Z_7^{N,\gamma}(t)}{N^{\max(\alpha_6,\alpha_7)}},\\
Z_{237}^{N,\gamma}(t) &\equiv \frac{N^{\alpha_2}Z_2^{N,\gamma}(t)+N^{\alpha_3}Z_3^{N,\gamma}(t)+N^{\alpha_7}Z_7^{N,\gamma}(t)}{N^{\max(\alpha_2,\alpha_3,\alpha_7)}},\\
Z_{679}^{N,\gamma}(t) &\equiv \frac{N^{\alpha_6}Z_6^{N,\gamma}(t)+N^{\alpha_7}Z_7^{N,\gamma}(t)+N^{\alpha_9}Z_9^{N,\gamma}(t)}{N^{\max(\alpha_6,\alpha_7,\alpha_9)}}.
\end{aligned}$$

The stochastic equations for the linear combinations of the species are given as

$$\begin{aligned}
Z_{23}^{N,\gamma}(t) &= Z_{23}^{N,\gamma}(0)+N^{-\max(\alpha_2,\alpha_3)}\Big[R_4^t(N^{\gamma+\rho_4}\kappa_4 Z_1^{N,\gamma})+R_8^t(N^{\gamma+\rho_8}\kappa_8 Z_7^{N,\gamma})\\
&\quad -R_9^t(N^{\gamma+\rho_9}\kappa_9 Z_2^{N,\gamma}Z_6^{N,\gamma})\Big],\\
Z_{67}^{N,\gamma}(t) &= Z_{67}^{N,\gamma}(0)+N^{-\max(\alpha_6,\alpha_7)}\Big[R_7^t(N^{\gamma+\rho_7}\kappa_7 Z_3^{N,\gamma})+R_{12}^t(N^{\gamma+\rho_{12}}\kappa_{12} Z_9^{N,\gamma})\\
&\quad -R_{10}^t(N^{\gamma+\rho_{10}}\kappa_{10} Z_6^{N,\gamma}Z_8^{N,\gamma})-R_{17}^t(N^{\gamma+\rho_{17}}\kappa_{17} Z_6^{N,\gamma})\Big],\\
Z_{237}^{N,\gamma}(t) &= Z_{237}^{N,\gamma}(0)+N^{-\max(\alpha_2,\alpha_3,\alpha_7)}\Big[R_4^t(N^{\gamma+\rho_4}\kappa_4 Z_1^{N,\gamma})-R_{15}^t(N^{\gamma+\rho_{15}}\kappa_{15} Z_4^{N,\gamma}Z_7^{N,\gamma})\Big],\\
Z_{679}^{N,\gamma}(t) &= Z_{679}^{N,\gamma}(0)+N^{-\max(\alpha_6,\alpha_7,\alpha_9)}\Big[R_7^t(N^{\gamma+\rho_7}\kappa_7 Z_3^{N,\gamma})-R_{17}^t(N^{\gamma+\rho_{17}}\kappa_{17} Z_6^{N,\gamma})\Big].
\end{aligned}$$

To show convergence of $Z_2^{N,2}$ and $Z_3^{N,2}$ as $N\to\infty$ in Section 5.1, we use an equation for $\big(\kappa_2 Z_2^{N,2}(t)-\kappa_3 Z_3^{N,2}(t)\big)^2$. Define

$$\mathcal{D}^{N,\gamma}(t) \equiv \frac{\kappa_2 N^{\alpha_2}Z_2^{N,\gamma}(t)-\kappa_3 N^{\alpha_3}Z_3^{N,\gamma}(t)}{N^{\max(\alpha_2,\alpha_3)}}.$$

Using the equations for $Z_2^{N,\gamma}$ and $Z_3^{N,\gamma}$ given in Section 1, $\mathcal{D}^{N,\gamma}$ satisfies

$$\begin{aligned}
\mathcal{D}^{N,\gamma}(t) &= \mathcal{D}^{N,\gamma}(0)+(\kappa_2+\kappa_3)N^{-\max(\alpha_2,\alpha_3)}\Big[R_3^t(N^{\gamma+\rho_3}\kappa_3 Z_3^{N,\gamma})\\
&\quad +R_5^t(N^{\gamma+\rho_5}\kappa_5 Z_3^{N,\gamma})+R_6^t(N^{\gamma+\rho_6}\kappa_6 Z_3^{N,\gamma})+R_7^t(N^{\gamma+\rho_7}\kappa_7 Z_3^{N,\gamma})\\
&\quad -R_2^t(N^{\gamma+\rho_2}\kappa_2 Z_2^{N,\gamma})\Big]+\kappa_2 N^{-\max(\alpha_2,\alpha_3)}\Big[R_4^t(N^{\gamma+\rho_4}\kappa_4 Z_1^{N,\gamma})\\
&\quad +R_8^t(N^{\gamma+\rho_8}\kappa_8 Z_7^{N,\gamma})-R_9^t(N^{\gamma+\rho_9}\kappa_9 Z_2^{N,\gamma}Z_6^{N,\gamma})\Big].
\end{aligned} \tag{1}$$

Using (1) and applying Ito's formula, we have

$$
\begin{aligned}
\mathcal{D}^{N,\gamma}(t)^2 &= \mathcal{D}^{N,\gamma}(0)^2 + N^{-\max(\alpha_2,\alpha_3)} \int_0^t 2(\kappa_2+\kappa_3)\mathcal{D}^{N,\gamma}(s-) \\
&\times \Big[dR_3^s(N^{\gamma+\rho_3}\kappa_3 Z_3^{N,\gamma}) + dR_5^s(N^{\gamma+\rho_5}\kappa_5 Z_3^{N,\gamma}) + dR_6^s(N^{\gamma+\rho_6}\kappa_6 Z_3^{N,\gamma}) \\
&+ dR_7^s(N^{\gamma+\rho_7}\kappa_7 Z_3^{N,\gamma}) - dR_2^s(N^{\gamma+\rho_2}\kappa_2 Z_2^{N,\gamma}) \Big] \\
&+ N^{-\max(\alpha_2,\alpha_3)} \int_0^t 2\kappa_2 \mathcal{D}^{N,\gamma}(s-) \Big[dR_4^s(N^{\gamma+\rho_4}\kappa_4 Z_1^{N,\gamma}) \\
&+ dR_8^s(N^{\gamma+\rho_8}\kappa_8 Z_7^{N,\gamma}) - dR_9^s(N^{\gamma+\rho_9}\kappa_9 Z_2^{N,\gamma} Z_6^{N,\gamma}) \Big] \\
&+ (\kappa_2+\kappa_3)^2 N^{-2\max(\alpha_2,\alpha_3)} \Big[R_3^t(N^{\gamma+\rho_3}\kappa_3 Z_3^{N,\gamma}) + R_5^t(N^{\gamma+\rho_5}\kappa_5 Z_3^{N,\gamma}) \\
&+ R_6^t(N^{\gamma+\rho_6}\kappa_6 Z_3^{N,\gamma}) + R_7^t(N^{\gamma+\rho_7}\kappa_7 Z_3^{N,\gamma}) + R_2^t(N^{\gamma+\rho_2}\kappa_2 Z_2^{N,\gamma}) \Big] \\
&+ \kappa_2^2 N^{-2\max(\alpha_2,\alpha_3)} \Big[R_4^t(N^{\gamma+\rho_4}\kappa_4 Z_1^{N,\gamma}) + R_8^t(N^{\gamma+\rho_8}\kappa_8 Z_7^{N,\gamma}) \\
&+ R_9^t(N^{\gamma+\rho_9}\kappa_9 Z_2^{N,\gamma} Z_6^{N,\gamma}) \Big].
\end{aligned} \tag{2}
$$

3 Scaling exponents and rate constants

Recall that the normalized rate constants are defined as

$$\kappa_k = \frac{\kappa'_k}{N_0^{\beta_k}}$$

where $N_0 = 100$ in this paper. In Table 2 and 3, unscaled and scaled rate constants are given with the corresponding scaling exponents.

Table 2: The unscaled stochastic reaction rate constants

	Rates		Rates		Rates
κ'_1	4.00×10^{0}	κ'_7	4.88×10^{-3}	κ'_{10}	3.62×10^{-4}
κ'_2	7.00×10^{-1}	κ'_8	4.40×10^{-4}	κ'_{11}	9.99×10^{-5}
κ'_3	1.30×10^{-1}	κ'_9	3.62×10^{-4}	κ'_{12}	4.40×10^{-5}
κ'_4	7.00×10^{-3}	κ'_{10}	3.62×10^{-4}	κ'_{13}	1.40×10^{-5}
κ'_5	6.30×10^{-3}	κ'_{11}	9.99×10^{-5}	κ'_{14}	1.40×10^{-6}
κ'_6	4.88×10^{-3}	κ'_{12}	4.40×10^{-5}	κ'_{15}	1.42×10^{-6}
κ'_7	4.88×10^{-3}	κ'_{13}	1.40×10^{-5}	κ'_{16}	1.80×10^{-8}
κ'_8	4.40×10^{-4}	κ'_{15}	1.42×10^{-6}	κ'_{17}	6.40×10^{-10}
κ'_9	3.62×10^{-4}	κ'_{16}	1.80×10^{-8}	κ'_{18}	7.40×10^{-11}

Table 3: The scaled stochastic reaction rate constants with scaling exponents

Scaled	rates	β_k		Scaled	rates	β_k		Scaled	rates	β_k	
κ_1	4	β_1	0	κ_7	0.488	β_7	-1	κ_{13}	0.14	β_{13}	-2
κ_2	0.7	β_2	0	κ_8	4.4	β_8	-2	κ_{14}	0.014	β_{14}	-2
κ_3	0.13	β_3	0	κ_9	3.62	β_9^* [1]	-2	κ_{15}	1.42	β_{15}^*	-3
κ_4	0.7	β_4	-1	κ_{10}	3.62	β_{10}^*	-2	κ_{16}	0.00018	β_{16}	-2
κ_5	0.63	β_5	-1	κ_{11}	0.999	β_{11}	-2	κ_{17}	0.0000064	β_{17}	-2
κ_6	0.488	β_6	-1	κ_{12}	0.44	β_{12}	-2	κ_{18}	0.00000074	β_{18}	-2

In Table 4, three sets of α_i's and ρ_k's we use in this model are given.

Table 4: Scaling exponents

α_i	First scaling	Second scaling	Third scaling	ρ_k	First scaling	Second scaling	Third scaling	ρ_k	First scaling	Second scaling	Third scaling
$\alpha_1^{\dagger}$ [2]	1	0	0	ρ_1	0	0	0	ρ_{10}	-2	-1	0
$\alpha_2^{\dagger}$	0	0	1	ρ_2	0	0	1	ρ_{11}	-2	-1	0
$\alpha_3^{\dagger}$	0	0	1	ρ_3	0	0	1	ρ_{12}	-2	-1	0
α_4	2	2	2	ρ_4	0	-1	-1	ρ_{13}	-2	-2	-2
α_5	2	2	2	ρ_5	-1	-1	0	ρ_{14}	-1	-2	-2
α_6	0	0	0	ρ_6	-1	-1	0	ρ_{15}	-1	-1	-1
α_7	0	0	0	ρ_7	-1	-1	0	ρ_{16}	0	0	0
$\alpha_8^{\dagger}$	0	1	2	ρ_8	-2	-2	-2	ρ_{17}	-2	-2	-2
$\alpha_9^{\dagger}$	0	1	2	ρ_9	-2	-2	-1	ρ_{18}	0	0	0

The initial species numbers of the parametrized family by N are given as

$$X_i^N(0) \equiv \left\lfloor \left(\frac{N}{N_0}\right)^{\alpha_i} X_i(0) \right\rfloor$$

and the normalized initial species numbers are given as

$$Z_i^{N,\gamma}(0) \equiv \frac{1}{N^{\alpha_i}} \left\lfloor \left(\frac{N}{N_0}\right)^{\alpha_i} X_i(0) \right\rfloor .$$

so that $Z_i^{N,\gamma}(0) \to \frac{1}{N_0^{\alpha_i}} X_i(0) \equiv Z_i^{\gamma}(0)$ as $N \to \infty$. In Table 5, the initial species numbers, $X_i(0)$, obtained from [1] and limits of the normalized initial species numbers, $Z_i^{\gamma}(0)$, in the three time scales are given.

[1] $*$ are scaling exponents for bimolecular reaction rate constants.
[2] α_i's depending on γ is marked by $\dagger$.

Table 5: Initial values used in the simulation

	Initial values	$\gamma = 0$	$\gamma = 1$	$\gamma = 2$
S_1	10	0.1	10	10
S_2	1	1	1	0.01
S_3	1	1	1	0.01
S_4	93	0.0093	0.0093	0.0093
S_5	172	0.0172	0.0172	0.0172
S_6	54	54	54	54
S_7	7	7	7	7
S_8	50	50	0.5	0.005
S_9	0	0	0	0

For the α_i's and ρ_k's determining the three scalings given in Table 4, we check the balance equations given in Table 4 in the main paper. If the balance equation fails, the corresponding time-scale constraint is computed. We have

Table 6: Balance conditions used in the model

	First scaling	Second scaling	Third scaling		First scaling	Second scaling	Third scaling
S_1	$\gamma \leq 2$	balanced	balanced	$S_2 + S_3 + S_7$	$\gamma \leq 0$	balanced	balanced
S_2	balanced	balanced	balanced	$S_2 + S_3$	$\gamma \leq 0$	$\gamma \leq 1$	balanced
S_3	balanced	balanced	balanced	$S_2 + S_7$	balanced	balanced	balanced
S_4	$\gamma \leq 2$	$\gamma \leq 2$	balanced	$S_6 + S_7 + S_9$	$\gamma \leq 1$	$\gamma \leq 2$	$\gamma \leq 2$
S_5	$\gamma \leq 2$	$\gamma \leq 2$	balanced	$S_6 + S_7$	$\gamma \leq 1$	balanced	balanced
S_6	$\gamma \leq 1$	balanced	balanced	$S_6 + S_9$	$\gamma \leq 1$	$\gamma \leq 2$	$\gamma \leq 2$
S_7	$\gamma \leq 1$	$\gamma \leq 1$	balanced	$S_8 + S_9$	$\gamma \leq 0$	$\gamma \leq 1$	balanced
S_8	$\gamma \leq 0$	$\gamma \leq 1$	balanced				
S_9	balanced	balanced	balanced				

4 Solution of balance conditions computed using Maple

To select values for scaling exponents based on the balance equations, we solve the balance equations with some conditions for monotonicity using *Maple*. The following gives the results we obtain from *Maple*.

$restart:$

$with(simplex)$

$BalanceEquations := \Big\{\beta_{13} = \alpha_1 + \beta_{14},$

$$\max(\alpha_3+\beta_3,\alpha_1+\beta_4,\alpha_3+\beta_5,\alpha_3+\beta_6,\alpha_3+\beta_7,\alpha_7+\beta_8)$$
$$=\max(\alpha_2+\beta_2,\alpha_2+\alpha_6+\beta_9),$$
$$\alpha_2+\beta_2=\max(\alpha_3+\beta_3,\alpha_3+\beta_5,\alpha_3+\beta_6,\alpha_3+\beta_7),$$
$$\alpha_3+\beta_6=\alpha_4+\beta_{18},\ \alpha_3+\beta_5=\alpha_5+\beta_{16},$$
$$\max(\alpha_3+\beta_7,\alpha_7+\beta_8,\alpha_9+\beta_{12},\alpha_4+\alpha_7+\beta_{15})$$
$$=\max(\alpha_2+\alpha_6+\beta_9,\alpha_6+\alpha_8+\beta_{10},\alpha_6+\beta_{17}),$$
$$\alpha_2+\alpha_6+\beta_9=\max(\alpha_7+\beta_8,\alpha_4+\alpha_7+\beta_{15}),$$
$$\max(\beta_1,\alpha_9+\beta_{12})=\max(\alpha_6+\alpha_8+\beta_{10},\alpha_8+\beta_{11}),$$
$$\alpha_6+\alpha_8+\beta_{10}=\alpha_9+\beta_{12},\ \alpha_1+\beta_4=\alpha_4+\alpha_7+\beta_{15},$$
$$\max(\alpha_1+\beta_4,\alpha_7+\beta_8)=\alpha_2+\alpha_6+\beta_9,$$
$$\max(\alpha_3+\beta_3,\alpha_1+\beta_4,\alpha_3+\beta_5,\alpha_3+\beta_6,\alpha_3+\beta_7)$$
$$=\max(\alpha_2+\beta_2,\alpha_4+\alpha_7+\beta_{15}),\ \alpha_3+\beta_7=\alpha_6+\beta_{17},$$
$$\max(\alpha_3+\beta_7,\alpha_7+\beta_8,\alpha_4+\alpha_7+\beta_{15})$$
$$=\max(\alpha_2+\alpha_6+\beta_9,\alpha_6+\beta_{17}),$$
$$\max(\alpha_3+\beta_7,\alpha_9+\beta_{12})=\max(\alpha_6+\alpha_8+\beta_{10},\alpha_6+\beta_{17}),$$
$$\beta_1=\alpha_8+\beta_{11}\Big\}$$

$$Conditions := \Big\{\beta_1-\beta_{13}\geq 0,\ \beta_9-\beta_{10}\geq 0,\ \beta_{10}-\beta_{15}\geq 0,\ \beta_2-\beta_3\geq 0,$$
$$\beta_3-\beta_4\geq 0,\ \beta_4-\beta_5\geq 0,\ \beta_5-\beta_6\geq 0,\ \beta_6-\beta_7\geq 0,$$
$$\beta_7-\beta_8\geq 0,\ \beta_8-\beta_{11}\geq 0,\ \beta_{11}-\beta_{12}\geq 0,\ \beta_{12}-\beta_{14}\geq 0,$$
$$\beta_{14}-\beta_{16}\geq 0,\ \beta_{16}-\beta_{17}\geq 0,\ \beta_{17}-\beta_{18}\geq 0\Big\}$$

$$Outputs := solve(BalanceEquations, useassumptions)\quad assuming\quad Conditions$$

$$\Big\{\alpha_1=-\beta_4+\alpha_4+\alpha_7+\beta_{15},\ \alpha_2=-\alpha_6-\beta_9+\max(\alpha_7+\beta_8,\alpha_4+\alpha_7+\beta_{15}),$$
$$\alpha_3=\alpha_3,\ \alpha_4=\alpha_4,\ \alpha_5=\alpha_3+\beta_5-\beta_{16},\ \alpha_6=\alpha_6,\ \alpha_7=\alpha_7,\ \alpha_8=\alpha_8,$$
$$\alpha_9=\alpha_6+\alpha_8+\beta_{10}-\beta_{12},\ \beta_1=\alpha_8+\beta_{11},$$
$$\beta_2=\alpha_6+\beta_9-\max(\alpha_7+\beta_8,\alpha_4+\alpha_7+\beta_{15})+\alpha_3+\beta_3,$$
$$\beta_3=\beta_3,\ \beta_4=\beta_4,\ \beta_5=\beta_5,\ \beta_6=-\alpha_3+\alpha_4+\beta_{18},\ \beta_7=-\alpha_3+\alpha_6+\beta_{17},$$
$$\beta_8=\beta_8,\ \beta_9=\beta_9,\ \beta_{10}=\beta_{10},\ \beta_{11}=\beta_{11},\ \beta_{12}=\beta_{12},$$

$$\beta_{13} = -\beta_4 + \alpha_4 + \alpha_7 + \beta_{15} + \beta_{14},\ \beta_{14} = \beta_{14},\ \beta_{15} = \beta_{15},\ \beta_{16} = \beta_{16},$$
$$\beta_{17} = \beta_{17},\ \beta_{18} = \beta_{18}\Big\}$$

5 The proof of Theorem 1

To prove the convergence for $\gamma = 0$ and $\gamma = 1$, we apply Theorem 4.1 in [2]. Following from Theorem 4.1 (and Remark 4.2) in [2], $Z^{N,\gamma} \Rightarrow Z^\gamma$ in the first time scale on $[0, \tau_\infty)$ where

$$\tau_\infty = \lim_{c\to\infty} \tau_c \equiv \inf\Big\{t : \sup_{s\le t} Z^\gamma(s) \ge c\Big\}.$$

The theorem is directly applicable for $\gamma = 0$, since the first time scale of interest in this model is when $\gamma = 0$. For $\gamma = 1$, we define a stopping time

$$\tau^1_{N,c} = \inf\Big\{t : \sup_{s\le t} Z^{N,1}(s) \ge c\Big\},$$

so that $Z^{N,1}(t)$ is bounded for $t \le \tau^1_{N,c}$. We compare a scaling exponent for each species number to those for all rates of reactions involving the species. Then, α_i is less or equal to $\gamma + \rho_k$ for each $k \in \Gamma_i^+ \cup \Gamma_i^-$ for all species except for Species 2 and 3, i.e., the only species possibly not bounded in this time scale are S_2 and S_3. On the other hand, $Z_2^{N,1}(t), Z_3^{N,1}(t) \le Z_{23}^{N,1}(t) \le c$ on $[0, \tau^1_{N,c})$. Therefore, relative compactness of $\{Z^{N,1}(\cdot \wedge \tau^1_{N,c})\}$ is satisfied, since all propensities $\hat\lambda_k(Z^{N,1}(\cdot \wedge \tau^1_{N,c}))$ are uniformly bounded. Then, $\big(Z^{N,1}(\cdot \wedge \tau^1_{N,c}), \tau^1_{N,c}\big) \Rightarrow \big(Z^1(\cdot \wedge \tau^1_c), \tau^1_c\big)$ for all but countably many c and we can set $\tau^1_\infty = \lim_{c\to\infty} \tau^1_c$.

5.1 The proof of the convergence for $\gamma = 2$

Proof. Computing natural time scales of the species, we get $\gamma_2 = \gamma_3 = \gamma_6 = 0$, $\gamma_7 = 1$, and $\gamma_1 = \gamma_4 = \gamma_5 = \gamma_8 = \gamma_9 = 2$. Since we already get the limiting models for S_2, S_3, S_{23}, S_6, S_7, and S_8 in the previous time scales, we set $\gamma = 2$ and derive a limiting model for a subset of species we are interested in. For $\gamma = 2$, $Z_1^{N,2}$, $Z_4^{N,2}$, $Z_5^{N,2}$, $Z_8^{N,2}$, and $Z_9^{N,2}$ are of order 1, and averaged behavior of $Z_2^{N,2}$, $Z_3^{N,2}$ $Z_6^{N,2}$, and $Z_7^{N,2}$ is expressed in terms of the limits of the scaled species numbers of order 1 in this time scale. In the section of limiting models in three time scales in the main text, we already derived limiting equations for Z_{23}^2, Z_8^2, and Z_9^2.

Define

$$\mathcal{D}^{N,2}(t) \equiv \kappa_2 Z_2^{N,2}(t) - \kappa_3 Z_3^{N,2}(t).$$

First, we will prove that the scaled species numbers for fast fluctuating species, S_2 and S_3, actually converge to a limit in a finite time interval. That is, for any fixed $\epsilon > 0$ and for any t such that $\epsilon < t \le \tau^2_\infty$,

$$Z_2^{N,2}(t) \longrightarrow \bar Z_2^2(t) = \frac{\kappa_3}{\kappa_2 + \kappa_3} Z_{23}^2(t), \tag{3}$$

$$Z_3^{N,2}(t) \quad \longrightarrow \quad \bar{Z}_3^2(t) = \frac{\kappa_2}{\kappa_2+\kappa_3} Z_{23}^2(t), \tag{4}$$

as $N \to \infty$ by showing $\mathcal{D}^{N,2}(t)^2 \to 0$ for $\epsilon < t \le \tau_\infty^2$ and using $Z_{23}^{N,2}(t) \to Z_{23}^2(t)$. The scaled species numbers of S_2 and S_3 may not converge to $\bar{Z}_2^2(t)$ and $\bar{Z}_3^2(t)$ in $t \in [0,\epsilon]$, since $\kappa_2 Z_2^{N,2}(0) - \kappa_3 Z_3^{N,2}(0)$ may not converge to zero. Plugging α_i's and ρ_k's in (2), we have

$$\begin{aligned}
&\mathcal{D}^{N,2}(t)^2 = \mathcal{D}^{N,2}(0)^2 + N^{-1}\int_0^t 2(\kappa_2+\kappa_3)\mathcal{D}^{N,2}(s-) \qquad (5)\\
&\times\Big[dR_3^s(N^3\kappa_3 Z_3^{N,2}) + dR_5^s(N^2\kappa_5 Z_3^{N,2}) + dR_6^s(N^2\kappa_6 Z_3^{N,2}) + dR_7^s(N^2\kappa_7 Z_3^{N,2}) - dR_2^s(N^3\kappa_2 Z_2^{N,2})\Big]\\
&+N^{-1}\int_0^t 2\kappa_2\mathcal{D}^{N,2}(s-) \times \Big[dR_4^s(N\kappa_4 Z_1^{N,2}) + dR_8^s(\kappa_8 Z_7^{N,2}) - dR_9^s(N\kappa_9 Z_2^{N,2} Z_6^{N,2})\Big]\\
&+N^{-2}(\kappa_2+\kappa_3)^2\Big[R_3^t(N^3\kappa_3 Z_3^{N,2}) + R_5^t(N^2\kappa_5 Z_3^{N,2}) + R_6^t(N^2\kappa_6 Z_3^{N,2}) + R_7^t(N^2\kappa_7 Z_3^{N,2}) + R_2^t(N^3\kappa_2 Z_2^{N,2})\Big]\\
&+N^{-2}\kappa_2^2\Big[R_4^t(N\kappa_4 Z_1^{N,2}) + R_8^t(\kappa_8 Z_7^{N,2}) + R_9^t(N\kappa_9 Z_2^{N,2} Z_6^{N,2})\Big].
\end{aligned}$$

Define reaction terms centered by their propensities as

$$\tilde{R}_k^t(N^{\gamma+\rho_k}\hat{\lambda}_k(Z^{N,\gamma})) \quad = \quad R_k^t(N^{\gamma+\rho_k}\hat{\lambda}_k(Z^{N,\gamma})) - \int_0^t N^{\gamma+\rho_k}\hat{\lambda}_k(Z^{N,\gamma}(s))\,ds.$$

Centering propensity in each reaction term in (5), we get

$$\mathcal{D}^{N,2}(t)^2 = \mathcal{D}^{N,2}(0)^2 + \mathcal{M}^N(t) + \int_0^t \left[-2N^2(\kappa_2+\kappa_3)\mathcal{D}^{N,2}(s)^2 + N\mathcal{E}^N(s)\right] ds, \tag{6}$$

where

$$\begin{aligned}
&\mathcal{E}^N(t) \equiv 2(\kappa_2+\kappa_3)(\kappa_5+\kappa_6+\kappa_7)\mathcal{D}^{N,2}(t-)Z_3^{N,2}(t)\\
&\quad +2N^{-1}\kappa_2\mathcal{D}^{N,2}(t-)\Big[\kappa_4 Z_1^{N,2}(t) + N^{-1}\kappa_8 Z_7^{N,2}(t) - \kappa_9 Z_2^{N,2}(t) Z_6^{N,2}(t)\Big]\\
&\quad +(\kappa_2+\kappa_3)^2\Big[\kappa_3 Z_3^{N,2}(t) + N^{-1}(\kappa_5+\kappa_6+\kappa_7)Z_3^{N,2}(t) + \kappa_2 Z_2^{N,2}(t)\Big]\\
&\quad +N^{-2}\kappa_2^2\Big[\kappa_4 Z_1^{N,2}(t) + N^{-1}\kappa_8 Z_7^{N,2}(t) + \kappa_9 Z_2^{N,2}(t) Z_6^{N,2}(t)\Big],
\end{aligned}$$

and

$$\begin{aligned}
&\mathcal{M}^N(t) \equiv N^{-1}\int_0^t 2(\kappa_2+\kappa_3)\mathcal{D}^{N,2}(s-)\\
&\quad\times\Big[d\tilde{R}_3^s(N^3\kappa_3 Z_3^{N,2}) + d\tilde{R}_5^s(N^2\kappa_5 Z_3^{N,2}) + d\tilde{R}_6^s(N^2\kappa_6 Z_3^{N,2}) + d\tilde{R}_7^s(N^2\kappa_7 Z_3^{N,2}) - d\tilde{R}_2^s(N^3\kappa_2 Z_2^{N,2})\Big]\\
&\quad +N^{-1}\int_0^t 2\kappa_2\mathcal{D}^{N,2}(s-) \times \Big[d\tilde{R}_4^s(N\kappa_4 Z_1^{N,2}) + d\tilde{R}_8^s(\kappa_8 Z_7^{N,2}) - d\tilde{R}_9^s(N\kappa_9 Z_2^{N,2} Z_6^{N,2})\Big]\\
&\quad +N^{-2}(\kappa_2+\kappa_3)^2\Big[\tilde{R}_3^t(N^3\kappa_3 Z_3^{N,2}) + \tilde{R}_5^t(N^2\kappa_5 Z_3^{N,2}) + \tilde{R}_6^t(N^2\kappa_6 Z_3^{N,2}) + \tilde{R}_7^t(N^2\kappa_7 Z_3^{N,2}) + \tilde{R}_2^t(N^3\kappa_2 Z_2^{N,2})\Big]\\
&\quad +N^{-2}\kappa_2^2\Big[\tilde{R}_4^t(N\kappa_4 Z_1^{N,2}) + \tilde{R}_8^t(\kappa_8 Z_7^{N,2}) + \tilde{R}_9^t(N\kappa_9 Z_2^{N,2} Z_6^{N,2})\Big].
\end{aligned}$$

In (6), $\left[-2N^2(\kappa_2+\kappa_3)(\mathcal{D}^{N,2})^2+N\mathcal{E}^N\right]$ is a drift and $\mathcal{M}^N$ gives noise of $(\mathcal{D}^{N,2})^2$ around its mean satisfying

$$E[\mathcal{M}^N(t)] \quad = \quad 0. \tag{7}$$

Taking an expectation in (6) and using (7), we get

$$E[\mathcal{D}^{N,2}(t)^2] \quad = \quad E[\mathcal{D}^{N,2}(0)^2] - \int_0^t 2N^2(\kappa_2+\kappa_3)E[\mathcal{D}^{N,2}(s)^2]\,ds + \int_0^t NE[\mathcal{E}^N(s)]\,ds. \tag{8}$$

By Gronwall's inequality, we get

$$E[\mathcal{D}^{N,2}(t)^2] \quad \leq \quad \Big(E[\mathcal{D}^{N,2}(0)^2] + \int_0^t NE[\mathcal{E}^N(s)]\,ds\Big)e^{-2N^2(\kappa_2+\kappa_3)t}. \tag{9}$$

Now, we will get an upper bound for the second moment of reaction terms. Let $X(\cdot)$ be a centered Poisson process with mean zero and τ be a stopping time for the process $\{X(t); t\geq 0\}$. Theorem 7 in [3] says that for $n \geq 2$, there exist constant C (finite and positive) depending on n such that

$$E[|X(\tau)|^n] \leq C\max\{E[\tau], E[\tau^{n/2}]\}. \tag{10}$$

Setting $\tau = \int_0^t N^{\gamma+\rho_k}\hat{\lambda}_k(Z^{N,\gamma}(s))\,ds$ and $X(\tau) = \tilde{R}_k^t(N^{\gamma+\rho_k}\hat{\lambda}_k(Z^{N,\gamma}))$, we have $E\left[X(\tau)\right]=0$ and Theorem 7 is applicable. Using Cauchy-Schwarz inequality, we get

$$\begin{aligned} E\big[R_k^t(N^{\gamma+\rho_k}\hat{\lambda}_k(Z^{N,\gamma}))^2\big] \quad &= \quad E\left[\left(\tilde{R}_k^t(N^{\gamma+\rho_k}\hat{\lambda}_k(Z^{N,\gamma})) + \int_0^t N^{\gamma+\rho_k}\hat{\lambda}_k(Z^{N,\gamma}(s))\,ds\right)^2\right] \\ &\leq 2E\left[\tilde{R}_k^t(N^{\gamma+\rho_k}\hat{\lambda}_k(Z^{N,\gamma}))^2\right] + 2E\left[\left(\int_0^t N^{\gamma+\rho_k}\hat{\lambda}_k(Z^{N,\gamma}(s))\,ds\right)^2\right]. \end{aligned} \tag{11}$$

Then, applying (10) to (11) and using Holder's inequality, we get an upper bound for the second moment of the random process.

$$\begin{aligned} &E\left[R_k^t(N^{\gamma+\rho_k}\hat{\lambda}_k(Z^{N,\gamma}))^2\right] \\ &\leq 2C_1E\left[\int_0^t N^{\gamma+\rho_k}\hat{\lambda}_k(Z^{N,\gamma}(s))\,ds\right] + 2E\left[\left(\int_0^t N^{\gamma+\rho_k}\hat{\lambda}_k(Z^{N,\gamma}(s))\,ds\right)^2\right] \\ &\leq 2C_1\int_0^t E\left[N^{\gamma+\rho_k}\hat{\lambda}_k(Z^{N,\gamma}(s))\right]\,ds + 2t\int_0^t E\left[N^{2(\gamma+\rho_k)}\hat{\lambda}_k(Z^{N,\gamma}(s))^2\right]\,ds \end{aligned} \tag{12}$$

Next, we will show $\sup_{t\leq\tau_\infty^2}\int_0^t E[\mathcal{E}^N(s)]\,ds < O(N)$ using boundedness of moments of reaction terms. Since $\mathcal{D}^{N,2}(t)^2 \leq (\kappa_2+\kappa_3)Z_{237}^{N,2}(t)^2$, using the equation for $Z_{237}^{N,2}(t)$ from the one in Section 2 and (12), we get

$$E[Z_{237}^{N,2}(t)^2] \quad \leq \quad 2E[Z_{237}^{N,2}(0)^2] + 2E\Big[\big(N^{-1}R_4^t(N\kappa_4Z_1^{N,2})\big)^2\Big]$$

$$\leq \quad 2E[Z_{237}^{N,2}(0)^2] + 4C_1 \int_0^t N^{-1}\kappa_4 E[Z_1^{N,2}(s)]\,ds + 4t\int_0^t \kappa_4^2 E[Z_1^{N,2}(s)^2]\,ds.$$

Using the equation for $Z_1^{N,2}(t)$ and (12), we get

$$\begin{aligned} E[Z_1^{N,2}(t)^2] &\leq 2E[Z_1^{N,2}(0)^2] + 2E\Big[R_{13}(\kappa_{13})^2\Big] \\ &\leq 2E[Z_1^{N,2}(0)^2] + 4C_1\kappa_{13}t + 4\kappa_{13}^2 t^2. \end{aligned}$$

Therefore,

$$\sup_N \sup_{t\leq \tau_\infty^2} E[Z_1^{N,2}(t)^2] \quad < \quad \infty. \tag{13}$$

and this gives

$$\sup_N \sup_{t\leq \tau_\infty^2} E[Z_{237}^{N,2}(t)^2] \quad < \quad \infty. \tag{14}$$

Using (14) and (8), we obtain

$$\sup_{t\leq\tau_\infty^2} \int_0^t E[\mathcal{E}^N(s)]\,ds \quad < \quad O(N),$$

and this and (9) imply that for any $\epsilon > 0$ independent of N and for $\tau_\infty^2 > \epsilon > 0$,

$$\begin{aligned} \sup_{\epsilon<t\leq\tau_\infty^2} E[\mathcal{D}^{N,2}(t)^2] \quad &\leq \quad \sup_{\epsilon<t\leq\tau_\infty^2} \Big(E[\mathcal{D}^{N,2}(0)^2] + \int_0^t NE[\mathcal{E}^N(s)]\,ds\Big)e^{-2N^2(\kappa_2+\kappa_3)t} \\ &\longrightarrow \quad 0, \end{aligned}$$

as $N \to \infty$.

Next, we will derive limiting equations for Z_1^2, Z_4^2, and Z_5^2. Using the equation for $Z_1^{N,\gamma}$ in Section 1, we get the equation for $Z_1^{N,2}$ as

$$Z_1^{N,2}(t) \quad = \quad Z_1^{N,2}(0) + R_{13}^t(\kappa_{13}) - R_{14}^t(\kappa_{14}Z_1^{N,2}).$$

Letting $N \to \infty$, we get

$$Z_1^2(t) \quad = \quad Z_1^2(0) + R_{13}^t(\kappa_{13}) - R_{14}^t(\kappa_{14}Z_1^2).$$

Since $Z_1^{N,2}(0) = Z_1^2(0) = X_1(0)$ due to $\alpha_1 = 0$, we actually have $Z_1^{N,2}(t) = Z_1^2(t)$. The equations for $Z_4^{N,2}$ and $Z_5^{N,2}$ are given from Section 1 as

$$\begin{aligned} Z_4^{N,2}(t) &= Z_4^{N,2}(0) + N^{-2}\Big[R_6^t(N^2\kappa_6 Z_3^{N,2}) - R_{18}^t(N^2\kappa_{18}Z_4^{N,2})\Big], \\ Z_5^{N,2}(t) &= Z_5^{N,2}(0) + N^{-2}\Big[R_5^t(N^2\kappa_5 Z_3^{N,2}) - R_{16}^t(N^2\kappa_{16}Z_5^{N,2})\Big]. \end{aligned}$$

Using the law of large numbers for Poisson processes and using (4), we get

$$\begin{aligned} Z_4^2(t) &= Z_4^2(0) + \int_0^t \left(\kappa_6 \bar{Z}_3^2(s) - \kappa_{18} Z_4^2(s)\right) ds, \\ Z_5^2(t) &= Z_5^2(0) + \int_0^t \left(\kappa_5 \bar{Z}_3^2(s) - \kappa_{16} Z_5^2(s)\right) ds. \end{aligned}$$

Now, we will show that $\int_0^t Z_2^{N,2}(s) Z_6^{N,2}(s)\, ds$, $\int_0^t Z_6^{N,2}(s)\, ds$, $\int_0^t Z_4^{N,2}(s) Z_7^{N,2}(s)\, ds$, and $\int_0^t Z_7^{N,2}(s)\, ds$ are stochastically bounded in a finite time interval. First, we show that $\int_0^t E[Z_6^{N,2}(s) Z_8^{N,2}(s)]\, ds$ and $\int_0^t E[Z_4^{N,2}(s) Z_7^{N,2}(s)]\, ds$ are bounded for $t \le \tau_\infty^2$. From the equation for $Z_{679}^{N,2}(t)$, we have

$$E[Z_{679}^{N,2}(t)] \quad \le \quad E[Z_{679}^{N,2}(0)] + \int_0^t \kappa_7 E[Z_3^{N,2}(s)]\, ds.$$

Since $\sup_{t \le \tau_\infty^2} E[Z_3^{N,2}(t)]$ is uniformly bounded due to (14), we get

$$\sup_N \sup_{t \le \tau_\infty^2} E[Z_{679}^{N,2}(t)] \quad < \quad \infty. \tag{15}$$

Using $N^{-2} E[Z_{67}^{N,2}(t)] \le E[Z_{679}^{N,2}(t)]$, we also get

$$\sup_N \sup_{t \le \tau_\infty^2} N^{-2} E[Z_{67}^{N,2}(t)] \quad < \quad \infty.$$

From the equation for $N^{-2} Z_{67}^{N,2}(t)$, we have

$$N^{-2} E[Z_{67}^{N,2}(t)] \quad \le \quad N^{-2} E[Z_{67}^{N,2}(0)] + \int_0^t \left(\kappa_7 E[Z_3^{N,2}(s)] + \kappa_{12} E[Z_9^{N,2}(s)] - \kappa_{10} E[Z_6^{N,2}(s) Z_8^{N,2}(s)]\right) ds.$$

Using (14) and (15), $\int_0^t \kappa_{10} E[Z_6^{N,2}(s) Z_8^{N,2}(s)]\, ds$ is uniformly bounded as

$$\begin{aligned} &\sup_N \sup_{t \le \tau_\infty^2} \int_0^t \kappa_{10} E[Z_6^{N,2}(s) Z_8^{N,2}(s)]\, ds \qquad (16) \\ \le\ &\sup_N \sup_{t \le \tau_\infty^2} \left\{ N^{-2} E[Z_{67}^{N,2}(0)] - N^{-2} E[Z_{67}^{N,2}(t)] + \int_0^t \left(\kappa_7 E[Z_3^{N,2}(s)] + \kappa_{12} E[Z_9^{N,2}(s)]\right) ds \right\} < \infty. \end{aligned}$$

Similarly from the equation for $Z_{237}^{N,2}(t)$, we have

$$E[Z_{237}^{N,2}(t)] \quad \le \quad E[Z_{237}^{N,2}(0)] + \int_0^t \left(\kappa_4 E[Z_1^{N,2}(s)] - \kappa_{15} E[Z_4^{N,2}(s) Z_7^{N,2}(s)]\right) ds.$$

Using (13), $\int_0^t \kappa_{15} E[Z_4^{N,2}(s) Z_7^{N,2}(s)]\, ds$ is uniformly bounded as

$$\begin{aligned} &\sup_N \sup_{t \le \tau_\infty^2} \int_0^t \kappa_{15} E[Z_4^{N,2}(s) Z_7^{N,2}(s)]\, ds \\ \le\ &\sup_N \sup_{t \le \tau_\infty^2} \left\{ E[Z_{237}^{N,2}(0)] - E[Z_{237}^{N,2}(t)] + \int_0^t \kappa_4 E[Z_1^{N,2}(s)]\, ds \right\} < \infty. \end{aligned}$$

Finally, we show stochastic boundedness of $\int_0^t Z_2^{N,2}(s)Z_6^{N,2}(s)\,ds$ and $\int_0^t Z_6^{N,2}(s)\,ds$ for $t \le \tau_\infty^2$. We split terms and obtain

$$\begin{aligned}
P\left(\int_0^t \kappa_9 Z_2^{N,2}(s)Z_6^{N,2}(s)\,ds > k\right) &\le P\left(\int_0^t \kappa_9 Z_6^{N,2}(s)Z_8^{N,2}(s)\,ds > \frac{k}{m}\right) + P\left(\sup_{t\le\tau_\infty^2} \frac{Z_2^{N,2}(t)}{Z_8^{N,2}(t)}\,ds > m\right) \\
&\le \underbrace{\frac{m}{k}E\left[\int_0^t \kappa_9 Z_6^{N,2}(s)Z_8^{N,2}(s)\,ds\right]}_{\text{(I)}} + \underbrace{P\left(\sup_{t\le\tau_\infty^2} \frac{Z_2^{N,2}(t)}{Z_8^{N,2}(t)}\,ds > m\right)}_{\text{(II)}}.
\end{aligned}$$

Using (16) and taking k large enough, we can make the term in (I) small. Since $Z_2^{N,2}$ and $Z_8^{N,2}$ converge to their limits as $N \to \infty$ and since $Z_8^{N,2}(0) \ne 0$, we can take m large to make the term in (II) small. Therefore, $\int_0^t Z_2^{N,2}(s)Z_6^{N,2}(s)\,ds$ is stochastically bounded. For $t \in [0, \tau_\infty^2]$ we have

$$\int_0^t 1_{[r,\infty)}(Z_6^{N,2}(s))\,ds \quad\le\quad \int_0^t \frac{Z_6^{N,2}(s)}{r}\,ds, \tag{17}$$

and taking the probability in both sides of (17), for fixed $\delta > 0$, we get

$$\begin{aligned}
P\left(\int_0^t \frac{Z_6^{N,2}(s)}{r}\,ds > \delta\right) &\le P\left(\inf_{t\le\tau_\infty^2} Z_8^{N,2}(t) \le \eta\right) + P\left(\int_0^t Z_6^{N,2}(s)Z_8^{N,2}(s)\,ds > r\delta\eta\right) \\
&\le \underbrace{P\left(\inf_{t\le\tau_\infty^2} Z_8^{N,2}(t) \le \eta\right)}_{\text{(III)}} + \underbrace{\frac{1}{r\delta\eta}E\left[\int_0^t Z_6^{N,2}(s)Z_8^{N,2}(s)\,ds\right]}_{\text{(IV)}}.
\end{aligned}$$

Since $Z_8^{N,2}(0) \ne 0$, we can take $\eta > 0$ small enough and r large enough to make both terms in (III) and (IV) small. Therefore, $Z_6^{N,2}$ is stochastically bounded for $t \in [0, \tau_\infty^2]$. Similarly, the stochastic boundedness of $Z_7^{N,2}$ can be shown using $\int_0^t Z_4^{N,2}(s)Z_7^{N,2}(s)\,ds$ and $\inf_{t\le\tau_\infty^2} Z_4^{N,2}(t)$ instead of $\int_0^t Z_6^{N,2}(s)Z_8^{N,2}(s)\,ds$ and $\inf_{t\le\tau_\infty^2} Z_8^{N,2}(t)$.

□

6 Sketch of the proof of Remark 3

Denote

$$\begin{aligned}
V_0^N(t) &= \left(Z_{23}^{N,2}(t), Z_4^{N,2}(t), Z_5^{N,2}(t), Z_8^{N,2}(t), Z_9^{N,2}(t)\right)^T, \\
V_0(t) &= \left(Z_{23}^2(t), Z_4^2(t), Z_5^2(t), Z_8^2(t), Z_9^2(t)\right)^T.
\end{aligned}$$

We already showed that $V_0^N \Rightarrow V_0$ as $N \to \infty$. We want to estimate an error between $V_0^N(t)$ and $V_0(t)$. Define $U^N(t) = r_N\left(V_0^N(t) - V_0(t)\right)$. If we show $U^N \Rightarrow U$, the error between $V_0^N(t)$ and $V_0(t)$ is approximately of order $r_{N_0}^{-1}$.

Suppose that $V_0^N(t)$ and $V_0(t)$ satisfy

$$M^{N,1}(t) = V_0^N(t) - V_0^N(0) - \int_0^t F^N(V^N(s))\,ds, \tag{18}$$

$$V_0(t) = V_0(0) + \int_0^t \bar{F}(V_0(s))\,ds, \tag{19}$$

where $F^N(V^N(t))$ is a drift and $M^{N,1}(t)$ is Poisson noise which gives fluctuations of V_0^N due to the corresponding reactions. V_0 is a solution of the stochastic processes whose randomness comes from Z_1^2. The drift term of V_0 is obtained from the drift term of V_0^N by replacing the species numbers fluctuating very rapidly by some variables describing their averaged behavior. Since Z_1^2 rarely moves during the time interval of our interest, V_0 behaves almost like a deterministic process. Denote A_N as a differential operator which gives instantaneous behavior of the normalized species numbers $Z^{N,2}$ during a very short time interval. For some function H_N which is identified later, $A_N H_N$ gives a drift for the process H_N and denote $M^{N,2}(t)$ as noise. Then, $M^{N,2}(t)$ satisfies

$$M^{N,2}(t) = H_N(V^N(t)) - H_N(V^N(0)) - \int_0^t A_N H_N(V^N(s))\,ds. \tag{20}$$

Adding (18) and (19) and multiplying by r_N, we get

$$r_N M^{N,1}(t) = U^N(t) - U^N(0) - r_N \int_0^t \left(F^N(V^N(s)) - \bar{F}(V_0(s))\right)\,ds. \tag{21}$$

Adding and subtracting terms, we rewrite (21) as

$$\begin{aligned} r_N M^{N,1}(t) = {} & U^N(t) - U^N(0) - r_N \int_0^t \left(\bar{F}(V_0^N(s)) - \bar{F}(V_0(s))\right)\,ds \\ & - r_N \int_0^t \left(F^N(V^N(s)) - F(V^N(s))\right)\,ds \\ & - r_N \int_0^t \left(F(V^N(s)) - \bar{F}(V_0^N(s))\right)\,ds. \end{aligned} \tag{22}$$

We identify H_N such that $A_N H_N \approx F - \bar{F}$, and using (20), (22) becomes

$$\begin{aligned} r_N M^{N,1}(t) \approx {} & U^N(t) - U^N(0) - r_N \int_0^t \left(\bar{F}(V_0^N(s)) - \bar{F}(V_0(s))\right)\,ds \\ & - r_N \int_0^t \left(F^N(V^N(s)) - F(V^N(s))\right)\,ds \\ & + r_N M^{N,2}(t) - r_N \left(H_N(V^N(t)) - H_N(V^N(0))\right). \end{aligned} \tag{23}$$

We can show that $r_N \int_0^t \left(F^N(V^N(s)) - F(V^N(s))\right)\,ds$ and $r_N\left(H_N(V^N(t)) - H_N(V^N(0))\right)$ converge to zero as N goes to infinity. We can show that $r_N\left(M^{N,1}(t) - M^{N,2}(t)\right) \Rightarrow M$ for an appropriately chosen r_N where

M is a process with mean-zero and independent increments satisfying

$$E\left[M(t)M^T(t)\right] = \int_0^t \bar{G}(V_0(s))\,ds.$$

If $\bar{G} = \sigma\sigma^T$, $U^N \Rightarrow U$ with U satisfying

$$U(t) = U(0) + \int_0^t \nabla\bar{F}(V_0(s))U(s)\,ds + \int_0^t \sigma(V_0(s))\,dW(s),$$

where $W(t)$ is a standard Brownian motion. Let $r_N = N^{1/2}$ and denote

$$U(t) = \left(U_{23}(t), U_4(t), U_5(t), U_8(t), U_9(t)\right)^T.$$

In the heat shock response model of *E. coli* for $\gamma = 2$, $U^N \Rightarrow U$ where U is a solution of

$$\begin{aligned} U(t) &= +\int_0^t \begin{bmatrix} 1 \\ 0 \\ 0 \\ 0 \\ 0 \end{bmatrix} \sqrt{\kappa_4 Z_1^2(s) + \kappa_9 \bar{Z}_2^2(s)\bar{Z}_6^2(s)}\,dW(s) \\ &+\int_0^t \begin{bmatrix} -\frac{\kappa_9}{\kappa_2+\kappa_3}\left(\kappa_3\bar{Z}_6^2(s) + \frac{\kappa_2\kappa_7}{\kappa_{10}}\cdot\frac{\bar{Z}_2^2(s)}{Z_8^2(s)}\right)U_{23}(s) + \kappa_9\frac{\bar{Z}_2^2(s)\bar{Z}_6^2(s)}{Z_8^2(s)}U_8(s) - \frac{\kappa_9\kappa_{12}}{\kappa_{10}}\cdot\frac{\bar{Z}_2^2(s)}{Z_8^2(s)}U_9(s) \\ \frac{\kappa_2\kappa_6}{\kappa_2+\kappa_3}U_{23}(s) - \kappa_{18}U_4(s) \\ \frac{\kappa_2\kappa_5}{\kappa_2+\kappa_3}U_{23}(s) - \kappa_{16}U_5(s) \\ -\frac{\kappa_2\kappa_7}{\kappa_2+\kappa_3}U_{23}(s) - \kappa_{11}U_8(s) \\ \frac{\kappa_2\kappa_7}{\kappa_2+\kappa_3}U_{23}(s) \end{bmatrix} ds. \end{aligned}$$

Noise of the error between V_0^N and V_0 comes from two sources: one from the Poisson noise of V_0^N due to the corresponding reactions and the other from a difference between the drift term of V_0^N and its averaged behavior. In the case for $\gamma = 2$, we find that the noise of the error mainly comes from the Poisson noise of V_0^N and is dominantly determined by the error between $Z_{23}^{N,2}(t)$ and $Z_{23}^2(t)$, since the species number of S_{23} has lower order of magnitude than those for S_4, S_5, S_8, and S_9. Errors are estimated using the central limit theorem derived in [4]. A detailed proof is omitted.

References

1. Srivastava R, Peterson M, Bentley W: **Stochastic kinetic analysis of the Escherichia coli stress circuit using σ^{32}-targeted antisense**. *Biotechnol Bioeng* 2001, **75**:120–129.
2. Kang HW, Kurtz T: **Separation of time-scales and model reduction for stochastic reaction networks** 2012. [To appear in Annals of Applied Probability (accepted). Online publication can be found in `http://www.imstat.org/aap/future_papers.html`. Preprint can be found in `http://arxiv.org/abs/1011.1672`].
3. Athreya K, Kurtz T: **A generalization of Dynkin's identity and some applications**. *The Annals of Probability* 1973, **1**(4):570–579.
4. Kang HW, Popovic L, Kurtz T: **Central limit theorems and diffusion approximations for multiscale Markov chain models** 2012. [Submitted. Preprint can be found in `http://arxiv.org/abs/1208.3783`].
